# The Correlation Between Three Teleconnections and Dengue Incidence in the Western Province of Sri Lanka, 2005–2019

**DOI:** 10.1029/2024GH001144

**Published:** 2025-09-23

**Authors:** N. D. B. Ehelepola, Kusalika Ariyaratne, R. M. P. Ratnayake

**Affiliations:** ^1^ Teaching (General) Hospital‐Peradeniya Peradeniya Sri Lanka; ^2^ Lanka Hydraulic Institute Moratuwa Sri Lanka; ^3^ National Hospital‐Kandy Kandy Sri Lanka

**Keywords:** public health, vector borne diseases, dengue, teleconnection, ENSO, ENSO modoki, Indian Ocean dipole, wavelet analysis

## Abstract

Dengue is an arboviral fever. Weather modulates dengue transmission by influencing the life cycles of vector mosquitoes and the virus. Three teleconnections are known to affect the weather in Sri Lanka. Those are El Nino Southern Oscillation (ENSO), Indian Ocean Dipole (IOD) and ENSO Modoki. We studied correlations between dengue incidence (DI) in the Western Province (WP) of Sri Lanka as a whole and three districts of the province and indices of ENSO, IOD and ENSO Modoki. We used four indices of ENSO and one index each of IOD and ENSO Modoki. We acquired notified dengue cases in WP, population data and monthly indices of three teleconnections for the 2005–2019 period. We used wavelet time series analysis to determine correlations between indices of teleconnections and DI. Two indices of ENSO were correlated with the DI of the WP and all three districts of the WP individually. The other two indices were correlated with the DI of two districts. The index of IOD was correlated with DI of two districts. The index of ENSO Modoki was correlated with the DI of WP and one district of it. Both positive and negative extremes of at least one teleconnection index were followed by the rise of DI in all districts. We concluded that three teleconnections modulate DI of different districts of WP in different ways. Monitoring of indices of these teleconnections and escalating dengue preventive work after extremes of indices can potentially blunt impending dengue peaks.

## Plain Language Summary

Dengue is a viral fever transmitted by Aedes mosquitoes. Weather modulates dengue transmission by influencing the life cycles of Aedes mosquitoes and the dengue virus. Teleconnections are irregularly recurring changes in the surface temperature of large areas of oceans coupled with corresponding changes in the lower atmosphere. Three teleconnections are known to modulate the weather in Sri Lanka. Those are El Nino Southern Oscillation (ENSO), Indian Ocean Dipole (IOD) and ENSO Modoki. Therefore, teleconnections are likely to modulate dengue transmission in Sri Lanka. The Western Province is the most seriously affected province of Sri Lanka by dengue. We studied correlations between indices of teleconnections and the dengue incidence in that province for 2005–2019. We used wavelet analysis method for that. Both positive and negative extremes of two teleconnections were followed by the rise of dengue in the province as a whole and in some districts of the province. Either positive or negative extremes of teleconnections were followed by rise of dengue in some others. Some teleconnections did not modify DI in some districts. Extremes of teleconnections are likely to occur more frequently with the on‐going climate changes. We describe the applicability of our results for dengue control.

## Key Points

- We have demonstrated that three teleconnections modulate the dengue incidence in the Western Province of Sri Lanka in two broad patterns
- Those teleconnections are El Nino Southern Oscillation (ENSO), Indian Ocean Dipole and ENSO Modoki
- Escalating dengue preventive work after extremes of teleconnections can potentially blunt impending dengue peaks in the province

## Graphical abstract

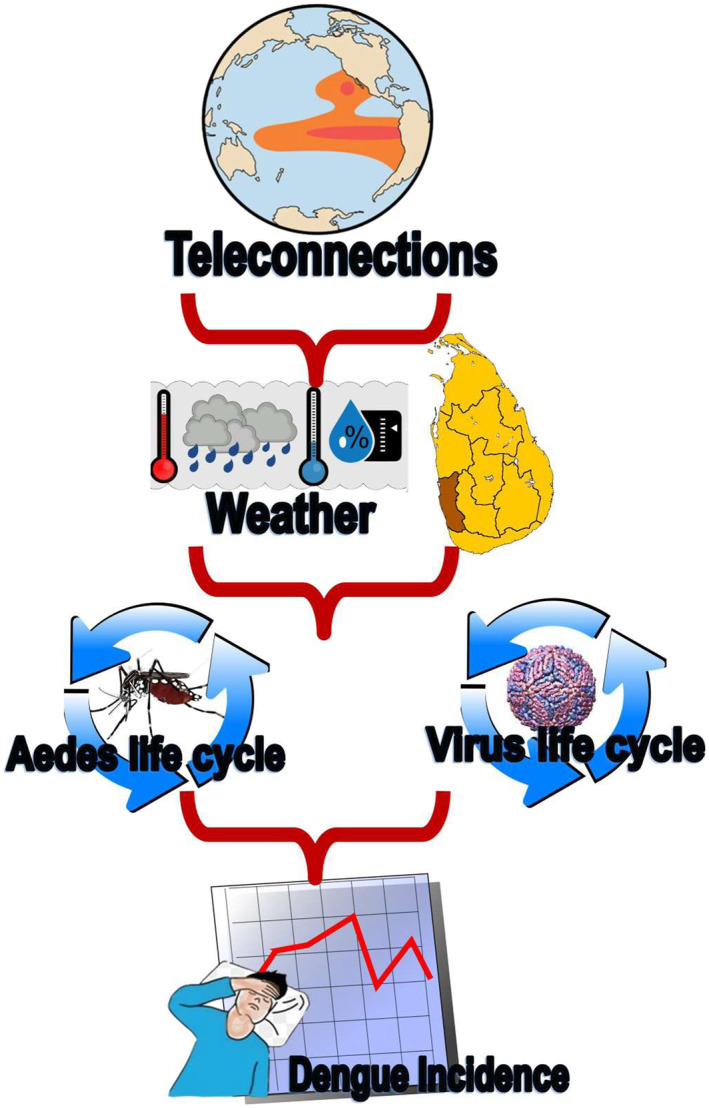

## 1 Introduction

Dengue is an emerging vector‐borne viral infection and one of the most important neglected tropical diseases (Ebi & Nealon, 2016; World Health Organization, 2024; Zeng et al., 2021). Out of >100 dengue endemic countries Sri Lanka is one of the most severely affected, with major outbreaks in years like in 2017 (Tissera et al., 2020). Western province (WP), one of the nine Provinces of the country, reports about 60% of dengue cases in Sri Lanka (Liyanage et al., 2016; Tissera et al., 2020). Thus optimizing dengue control of WP is a pressing priority.

The life cycles of both dengue vectors (*Aedes aegypti* and *Aedes albopictus*) and virus as well as the biting habits of *Aedes* mosquitoes are modified by weather (Abdulla et al., 2022; Bellone & Failloux, 2020; Brown et al., 2023; Ebi & Nealon, 2016; Ezeakacha & Yee, 2019). Sri Lanka's weather is modulated by teleconnection (Abeysekera et al., 2021; Hapuarachchi & Jayawardene, 2018; Zubair et al., 2003). All things considered, understanding how teleconnections modulate the DI of WP will be useful to fine‐tune dengue control work of the WP to achieve better results.

In subtopic 1.1, we explain how weather parameters affect the life cycle of the Aedes vector and dengue transmission, and in subtopic 1.2, we describe teleconnections and how do they influence the weather in Sri Lanka. Subtopic 1.3. Illustrates the significance of the dengue burden in the WP. That background knowledge would be helpful to appreciate how teleconnections modulate the DI of WP and the importance of this study.

### 1.1 How Weather Parameters Affect the Life Cycle of the *Aedes* Vector and Dengue Transmission

*Aedes* are primarily container breeders (Abdullah et al., 2022; Ebi & Nealon, 2016). Rainfall affects mosquito abundance by creating breeding sites, stimulating hatching of existing *Aedes* eggs when rainwater immerses eggs and by topping up water levels in containers until eggs become adults (Abdullah et al., 2022; Ebi & Nealon, 2016). However, during heavy downpours, *Aedes* larvae and pupae can get flushed out and the flight of adult *Aedes* also be hindered (Abdullah et al., 2022; Ebi & Nealon, 2016). An increase in temperatures upsurges the reproductive rate and biting frequency of *Aedes* (Abdulla et al., 2022; Ebi & Nealon, 2016). That also shortens the egg‐to‐ adult time period of *Aedes*, the incubation period of the virus inside the mosquito's body and increases the risk of dengue transmission to people per bite (Abdulla et al., 2022; Bellone & Failloux, 2020; Ebi & Nealon, 2016). The temperature to which larvae were exposed modulates adult *Aedes* fecundity (Ebi & Nealon, 2016). The best temperature range for *Aedes* survival and optimum extrinsic incubation period is 18–31°C (Abdulla et al., 2022). Wide diurnal temperature ranges (DTRs) lengthen the part of the *Aedes* life cycle in water, reduce the reproductive output of female mosquitoes, curtail the lifespans of *Aedes*, make them less susceptible to dengue virus infections and lessen the risk of dengue transmission to people per bite (Ebi & Nealon, 2016; Ehelepola & Ariyaratne, 2016). Humidity affects the sustainability of breeding sites, flight, lifespan and reproduction activities of *Aedes* (Abdulla et al., 2022; Brown et al., 2023; Ebi & Nealon, 2016). Strong winds disperse mosquitoes and make it difficult for them to find a host, yet they help extend the range of mosquitoes (Gu et al., 2016). Sunshine intensity/cloud cover influences the biting habits of *Aedes* (Ehelepola et al., 2015; Gu et al., 2016).

### 1.2 What Are Teleconnections and How Do They Influence on Weather in Sri Lanka

Teleconnections are important links between weather phenomena at spatially widely separated locations on earth, which typically involve climate patterns that span thousands of kilometers (Liu & Alexander, 2007; Zavadoff & Arcodia, 2022). Three teleconnections are known to affect the weather in Sri Lanka. Those are ENSO, IOD (also known as Indian Ocean Zonal mode) and ENSO Modoki (Abeysekera et al., 2021; Hapuarachchi & Jayawardene, 2018; Zubair et al., 2003). An index of a teleconnection is an indicator of the phase and strength of that teleconnection (McGregor & Ebi, 2018). ENSO is a triphasic oscillation of sea surface temperature (SST) in the Eastern and Western sides of the tropical Pacific Ocean and corresponding atmospheric pressure fluctuations known as Southern oscillation (Liu & Alexander, 2007; McGregor & Ebi, 2018; Zubair et al., 2003). That gives rise to changes in airflow patterns in Walker circulation, modulating weather in many parts of the world including Sri Lanka (Liu & Alexander, 2007; McGregor & Ebi, 2018; Zubair et al., 2003). ENSO is one of the most important modulators of inter‐annual climate variability (McGregor & Ebi, 2018). Hence, ENSO is associated with climate related adverse health outcomes worldwide (McGregor & Ebi, 2018). When the SST of the Eastern Tropical Pacific Ocean is higher than a threshold level, it is called El Nino (positive = warm phase of ENSO), the opposite is called La Nina (negative = cold phase) and there is an intermediate neutral phase (McGregor & Ebi, 2018; Zubair et al., 2003). In this study, El Ninos plus positive phase extremes not strong enough to be labeled as El Ninos are considered as positive phase extremes. Nino indices are indicators of the phase and strength of ENSO (McGregor & Ebi, 2018). Nino 3.4 and Nino 4 are two commonly used indices of SST anomalies (SSTA) in ENSO (Ashok et al., 2007; McGregor & Ebi, 2018). The index of atmospheric pressure fluctuations of ENSO is the Southern Oscillation Index (SOI) (McGregor & Ebi, 2018). There are two widely used versions of the SOI (McGregor & Ebi, 2018). We use the Equatorial Southern Oscillation Index (EQSOI) for the present study. The Multivariate ENSO Index (MEI) is a blended index merging five variables of both SST and atmospheric components of the ENSO (McGregor & Ebi, 2018).

IOD is a coupled ocean atmosphere phenomenon related to the tropical Indian Ocean (Behera et al., 2006; Zubair et al., 2003). Only SSTA indices are available to the public for studies regarding IOD and ENSO Modoki. Lower SST in the South Eastern part of the tropical Indian Ocean than the Western part and corresponding changes in atmospheric pressure are the positive phase of IOD and vice versa is the negative phase (Behera et al., 2006; Zubair et al., 2003). The index of SSTA of the IOD is called the Dipole Mode Index (DMI) (Abeysekera et al., 2021; Behera et al., 2006).

ENSO Modoki also has positive, neutral and negative phases (Ashok et al., 2007; Hapuarachchi & Jayawardene, 2018). During its positive phase (El Nino Modoki) the central equatorial Pacific Ocean becomes warmer with colder Western and Eastern flanks and coupled atmospheric pressure changes (Ashok et al., 2007; Hapuarachchi & Jayawardene, 2018). During the negative phase (La Nina Modoki) the central equatorial Pacific Ocean becomes colder with warmer Western and Eastern flanks (Ashok et al., 2007; Hapuarachchi & Jayawardene, 2018). El Nino Modoki Index (EMI), calculated reflecting these SSTA is the index of the ENSO Modoki (Ashok et al., 2007; Hapuarachchi & Jayawardene, 2018).

Sometimes those three teleconnections interact with each other in modulating the weather in Sri Lanka (Abeysekera et al., 2019; Burt & Weerasinghe, 2014). Teleconnections interact with other atmospheric phenomena like monsoon winds and modulate weather in Sri Lanka including WP (Abeysekera et al., 2019; Burt & Weerasinghe, 2014; Hapuarachchi & Jayawardene, 2018).

### 1.3 The Significance of the Dengue Burden in the WP

Dengue prevention and control work was enhanced in Sri Lanka especially in the WP, during our period of study and there has been a presidential task force chaired by the head of state since 2010 on this subject, unlike for any other infectious disease. Despite the priority given for dengue control, the disease burden of dengue in the WP has been rising and is very high compared to many other nations, according to real world data. We found that during 2013–2019, there were on average 27,000 dengue cases reported from the most populous province of Sri Lanka WP annually, including 71,619 cases reported in the outbreak year 2017 (Epidemiology Unit 2005–2019). During recent outbreaks, most dengue cases and deaths in Sri Lanka were among working age people. Outbreaks like that of 2017 cause high morbidity and mortality and controlling outbreaks are costly (Malavige et al., 2021; Tissera et al., 2020). For example, direct expenditure by the government on dengue control in Sri Lanka was US $12.7 million in 2017 (Tissera et al., 2020).

### 1.4 Why We Did This Study

Generally, there is a paucity of long term studies on how multiple teleconnections modulate the DI of dengue endemic regions, especially in South Asia including Sri Lanka. The authors of the latest published study from South Asia on teleconnection dengue correlation conducted their study at the State level of India, which is larger than WP (Pramanik et al., 2020). In their conclusion, they highlighted the necessity of future similar studies in smaller areas, combining the index of IOD (Pramanik et al., 2020). To the best of our knowledge, the present study is the first to determine the influence of ENSO Modoki on DI in South Asia. We found only one study determining the correlation between ENSO Modoki and DI from anywhere in English literature (Yip et al., 2022). To our knowledge, there are no past studies that simultaneously used SSTA indices, an atmospheric index and a blended index of ENSO to study ENSO versus DI correlation. If a researcher uses an index of only one category he or she might miss the identification of a truly existing correlation. Using all three categories of indices for a study is helpful to avoid this and get a more reliable idea of the correlation. Both ENSO and IOD have been demonstrated to modulate monsoon winds and rainfall in Sri Lanka (Abeysekera et al., 2021; Hapuarachchi & Jayawardene, 2018; Zubair et al., 2003). ENSO Modoki also known to modulate rainfall in Sri Lanka (Hapuarachchi & Jayawardene, 2018). Out of nine Provinces of Sri Lanka, WP reported the highest number of cases and deaths due to dengue 2005–2019 (Epidemiology Unit 2005–2019). Past studies conducted in the WP show that DI is correlated with local weather parameters like rainfall, temperature and humidity (Ehelepola & Ariyaratne, 2016; Liyanage et al., 2016)). All in all, mechanistically there are good reasons to expect DI of WP to be correlated with ENSO, ENSO Modoki and IOD. There is no monotonic correlation pattern between Sri Lankan weather and teleconnections. For example, one study shows El Nino gives rise to higher rainfall during October to December but declines the rainfall in January and February in Sri Lanka (Burt & Weerasinghe, 2014). Teleconnections do not modulate rainfall (weather) uniformly within WP of Sri Lanka (Abeysekera et al., 2021). Consequently, we decided to find correlations of DI of three districts of the WP with the six indices of teleconnections separately as well. Considering all the aforementioned, the importance of dengue prevention is obvious. Revisiting dengue epidemiology in Sri Lanka especially in the worst affected Province WP and detecting factors that influence local DI, ranking them and modifying dengue preventive work accordingly, is a need of the hour.

## 2 Materials and Methods

### 2.1 Study Setting

**Figure 1:**
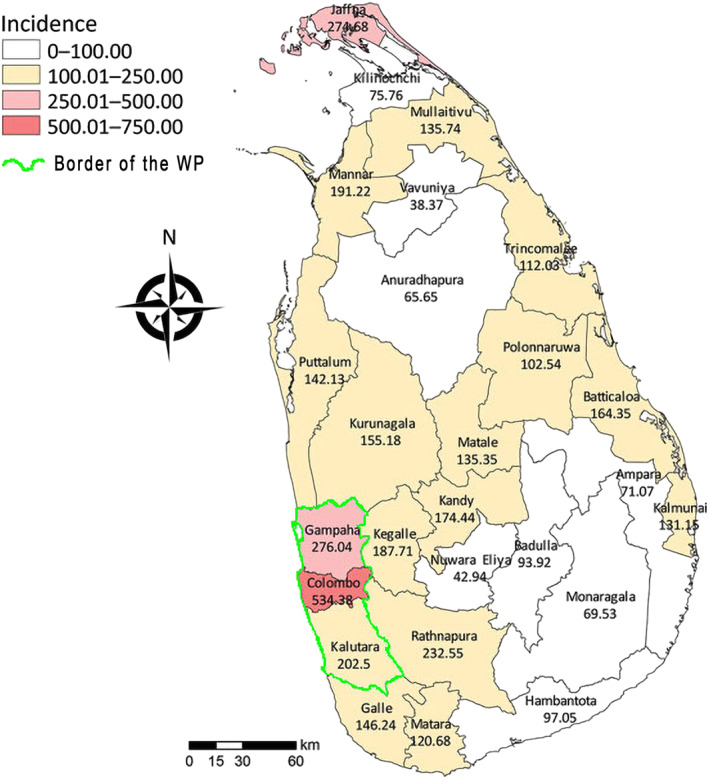
This map portrays districts of Sri Lanka. The border of our area of study, Western Province (WP) is marked by a green color line. WP is made up of three districts: Gampaha, Colombo and Kalutara. The mean dengue incidence per 100,000 inhabitants in each district from 2012 to 2016 is shown in figures and each district is color coded accordingly. This map is a modification of the map, Figure 2 of our reference (Tissera et al., 2020), created under CC BY4.0.

WP is situated in the west coast lowlands of Sri Lanka and has an area of 3,684 km^2^ and comprising Colombo, Gampaha and Kalutara districts. All three districts belong to the wet climatic zone of Sri Lanka. Both the largest city, which is also the commercial capital, plus the administrative capital of the nation are in Colombo district. The estimated population of the WP in 2005 and 2019 respectively, was 5.61 million and 6.15 million. Our study area is shown on a map of Sri Lanka in Figure 1.

### 2.2 Objectives and Hypotheses

Our main objective was to find the correlation between ENSO (represented by Nino 3.4, Nino4, EQSOI and MEI indices), IOD (represented by DMI), ENSO Modoki (represented by EMI) and DI of the WP and three districts of the WP for 2005–2019. Our secondary objective was to compare our results with those of related and similar studies ((Atique et al., 2016; Banu et al., 2015; Chuang et al., 2017; Earnest et al., 2012; Ehelepola, Ariyaratne, & Dissanayake, 2021; Imai et al., 2016; Kakarla et al., 2019; Liyanage et al., 2016, 2022; Pramanik et al., 2020).

We explored potential ways to improve local dengue control using the information generated and other available evidence as well. Our hypothesis was that teleconnections modulate the DI of the WP.

### 2.3 Data

We obtained the counts of dengue cases notified from WP each week from the weekly epidemiology reports of the Ministry of Health of Sri Lanka from 2005 to 2019 (Epidemiology Unit 2005–2019). Notified dengue case numbers for weeks 26 and 31 of 2005 and week 3 of 2006 were missing. Only three weeks of data were missing for entire period of 2005–2019 (0.4%). The missing weeks were filled with linear interpolation, considering the nearest available data. The annual estimated mid‐year population of the WP for 2005–2019 period was obtained from the Sri Lanka Department of Census and Statistics. Monthly Nino 3.4, Nino 4, EQSOI index, plus monthly DMI were obtained online from the National Oceanic and Atmospheric Administration of the United States and monthly EMI data from the Japan Agency for Marine‐Earth Science and Technology. There was no missing data in the mid‐year population and teleconnection indices. Our data sharing statement gives further details.

### 2.4 Analysis

We have estimated weekly DI per 100,000 population for the 2005–2019 period. We converted weekly DI to monthly DI for analysis. We determined correlation patterns with lag periods between indices of teleconnections and monthly DI by wavelet analysis.

The correlation between weather parameters/teleconnection indices and dengue is also nonlinear and non‐stationary (Ehelepola & Ariyaratne, 2016). Wavelet time series analysis (wavelet analysis) is very suitable to detect nonlinear and non‐stationary correlations like those between teleconnection indices and weather sensitive infectious diseases incidences and was used in similar past studies by us and others (Banu et al., 2015; Ehelepola & Ariyaratne, 2016). Moreover, wavelet analysis can differentiate multi‐annual patterns of variation from strong seasonal variation, and frequency components of different time series can be compared directly in wavelet coherence (Johansson et al., 2009).

#### 2.4.1 Wavelet Analysis

We performed wavelet analysis in a very similar way to similar publications by us (Ehelepola, Ariyaratne, & Dissanayake, 2021; Ehelepola et al., 2015).

The majority of mathematical techniques, including Fourier analysis, which investigate periodicities in the frequency domain unreservedly presume that the process is time stationary. Wavelet transforms, on the other hand, extend time series into time‐frequency space and can locate localized intermittent periodicities as a result. In wavelet analysis, an appropriate window is selected, moved along the signal, and the spectrum is computed at every position. This process is iterated repeatedly, with each subsequent cycle involving a slightly longer and shorter window. A set of time‐frequency representations of the signal with varying resolutions is what the wavelet transform produces. Because it could be utilized to analyze time series with non‐stationary power at different frequencies, the wavelet transform is significant. A time series can be broken down into time‐frequency space to identify the primary modes of variability and the temporal variations of those modes. Two techniques are used to analyze the correlation between two time series in time frequency space: cross wavelet transform (XWT) and wavelet coherence (WTC). Even though XWT is frequently used to analyze localized intermittent oscillations in time series, it is also useful to look at two time series that are possibly related. Specifically, to investigate if areas in the time frequency space with high common powers exhibit a stable phase relationship, indicating a causal relationship between those time series. We generated the continuous wavelet transform (CWT) for the meteorological variable under investigation. Using the wavelet as a bandpass filter on the time series is the concept underlying the CWT. The wavelet is stretched in time by varying its scale, s, so that η = st, and normalizing it to have unit energy. η is dimensionless time.

The CWT of a time series, $X_{n}$, *n* = 1,2,…,N with uniform time step δt, is defined as the convolution of $X_{n}$ with the scaled and normalized wavelet (Grinsted et al., 2004; Torrence & Compo, 1998).

$$W_{n}^{X}\left(s\right)=\sqrt{\frac{\delta t}{s}}\sum_{n^{\prime }=1}^{N}X_{n^{\prime }}\psi_{0}\left[\left(n^{\prime }-n\right)\frac{\delta t}{s}\right]$$

In the present analysis, Morlet wavelet was used. When using wavelets for feature extraction purposes, the Morlet wavelet with $\omega_{0}$ = 6 is a good choice, since it provides a good balance between time and frequency localization (Jevrejeva et al., 2003).

Wavelet power (Grinsted et al., 2004):

$${\left|W_{n}^{X}\left(s\right)\right|}^{2}$$

When the DI CWT was compared to teleconnection indices, it was evident that there were comparable features in the wavelet power. To investigate the potential for shared power, the cross‐wavelet transform was used. Regions in time frequency space where the time series exhibit significant common power are identified via the Cross Wavelet Transform (XWT). The cross wavelet transform of two time series $X_{n}$ and $Y_{n}$ is defined as (Grinsted et al., 2004):

$$W^{XY}=W^{X}W^{Y*},$$

where * denotes complex conjugation.

Cross wavelet power = $\left|W^{\text{XY}}\right|$ (Grinsted et al., 2004).

To determine whether there could be a causality impact, the wavelet coherence (WTC) was computed. The square of the cross spectrum normalized by the individual power spectra is the definition of wavelet coherence. This calculates the cross‐correlation between two time series as a function of frequency and returns a value between 1 and 0. It indicates causation between time series if there are areas in time frequency space with substantial common power and a constant phase connection (Grinsted et al., 2004).

Wavelet coherence (Grinsted et al., 2004):

$$R_{n}^{2}\left(s\right)=\frac{{\left|S\left(s^{-1}W_{n}^{XY}\left(s\right)\right)\right|}^{2}}{S\left(s^{-1}{\left|W_{n}^{X}\left(s\right)\right|}^{2}\right).S\left(s^{-1}{\left|W_{n}^{Y}\left(s\right)\right|}^{2}\right)}$$

*S* is a smoothing operator.

Reconstructing the time series for the period that yields the most power allowed us to determine the leading or lagging time. We could reconstruct the original time series since the wavelet transform is a band pass filter with a known wavelet function.

Reconstructed time series (Grinsted et al., 2004):

xn=δjδt12Cδψ0(0)∑j=0JRWnsjsj12

In this equation, $\psi_{0}\left(0\right)$ removes the energy scaling and sj12 converts the wavelet transform to an energy density. For each wavelet function the factor $C_{\delta }$ is a constant (Torrence & Compo, 1998).

We created a wavelet‐filtered time series by taking into account the aforementioned equation and summing over a portion of the scales. The analysis determined the times with the strongest coherence between the indices of teleconnections and the DI. We computed the lagging times and rebuilt the wavelet‐filtered time series for the research period. MATLAB Corporation's (USA) R2013a program was used to do the wavelet analysis.

Ethical clearance was given by the ethics review committee of the Faculty of Medicine, University of Peradeniya, Sri Lanka (2021/EC/57).

## 3 Results

We present our results in a similar manner as in our past studies done using the same methodology (Ehelepola & Ariyaratne, 2016; Ehelepola, Ariyaratne, & Dissanayake, 2021).

During 2005–2019, annual DI of the WP was lowest in 2008 (45.2/100,000) and highest in 2017 (1177.8/100,000).

### 3.1 Time Series Graphs of the DI of the WP and Indices of Three Teleconnections, 2005–2019

**Figure 2:**
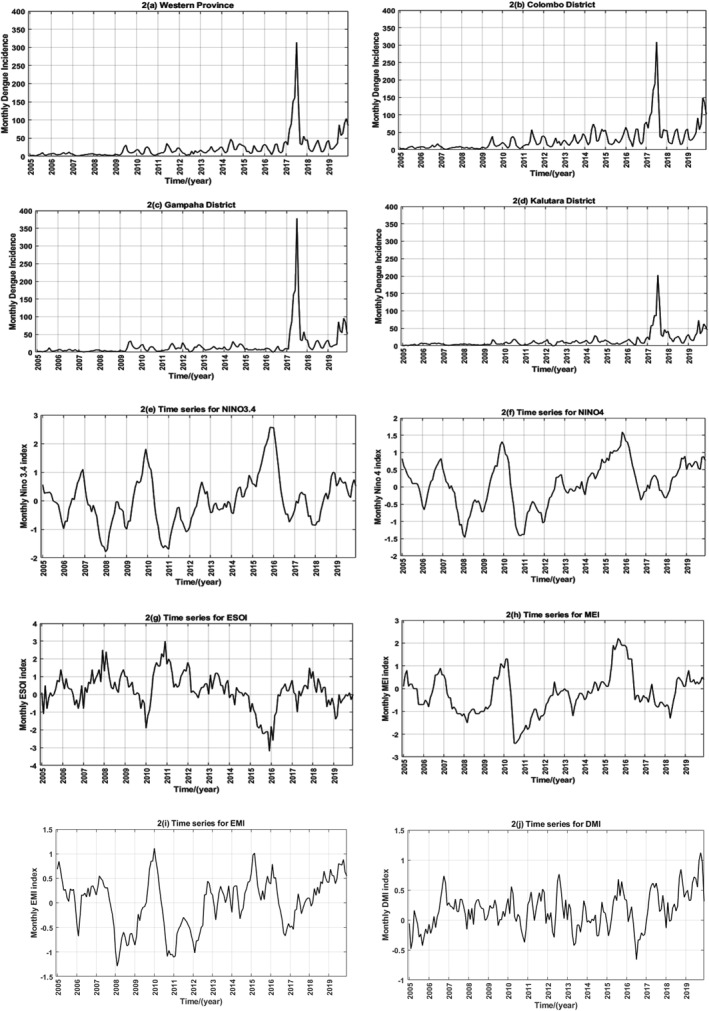
Time series graphs showing the three teleconnections' indices and the incidence of dengue during the study period. 2a–2d are time series graphs of the monthly dengue incidence (DI) (per 100,000 population) for 2005–2019. *x*‐axis of 2a–2d: time (year); *y*‐axis: monthly DI (per 100,000 population). 2a: Western Province; 2b: Colombo district; 2c: Gampaha district; 2d: Kalutara district. 2e–2i are time series graphs of the six teleconnection indices for 2005–2019. *x*‐axis of 2d–2i: time (year); *y*‐axis: monthly index of the teleconnection. 2e: NINO3.4, 2f: NINO 4, 2g: ESOI, 2h: MEI, 2i: EMI, 2j: DMI.

We present time series graphs of the DI of the WP and indices of three teleconnections, 2005–2019, as Figure 2.

DI of the WP and three districts of the WP showed a rising trend during 2005–2019 especially after 2009. A peak of DI in 2017 is obvious in WP as well as in all districts of it. The DI of Colombo is generally higher than the other two districts. Figures 2e–2j illustrate how indices of teleconnections fluctuated. Temporal changes in Nino 3.4 and Nino 4 (Figures 2e and 2j) show very similar patterns.

### 3.2 Wavelet Analysis Results

**Figure 3:**
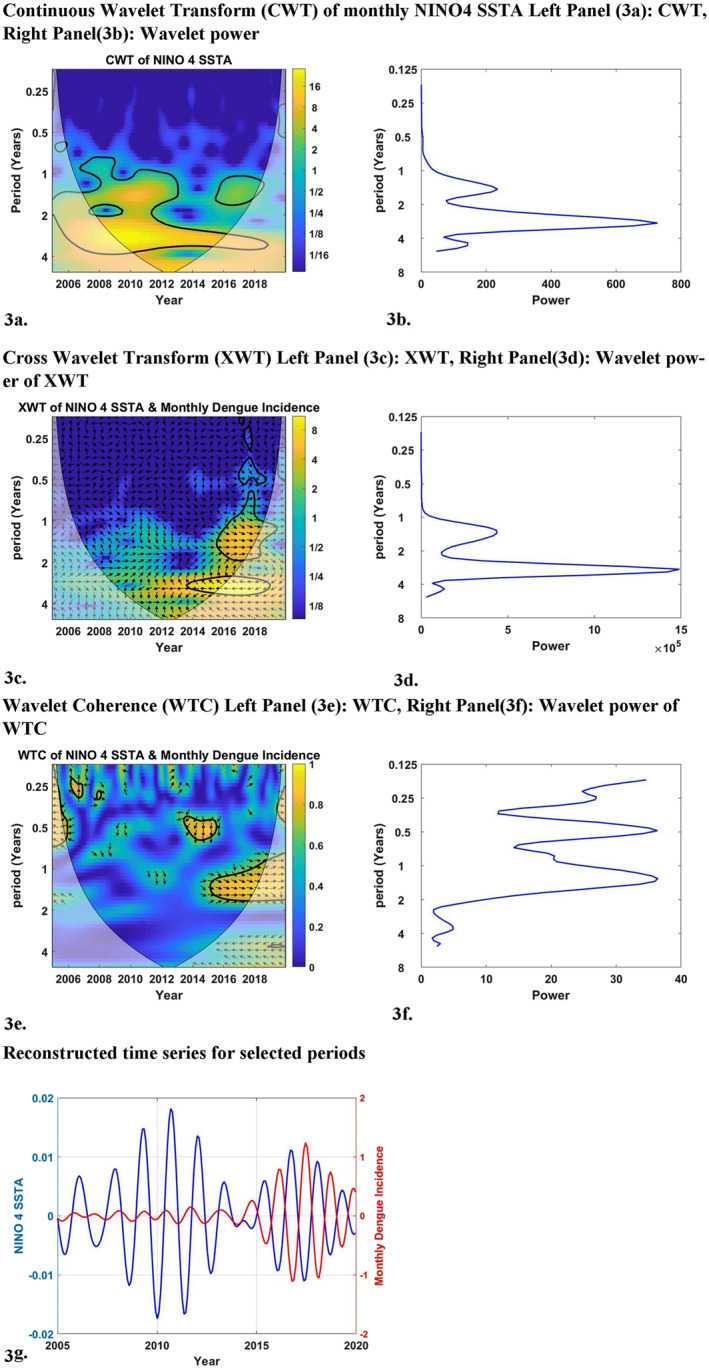
Wavelet analysis results of monthly Nino 4 SSTA versus dengue incidence for 2004–2019 (our study period): Panel 3a shows continuous wavelet transform (CWT) variations; Panel 3b shows wavelet power of CWT; Panel 3c shows cross wavelet transform (XWT) variations; Panel 3d shows wavelet power of XWT; Panel 3e shows wavelet coherence (WTC); Panel 3f shows wavelet power of WTC; Panel 3g shows reconstructed time series for 2005–2019.

We present Figure 3 as a sample of our wavelet analysis results. Figure 3 illustrates the results of the wavelet analysis of monthly Nino4 anomaly versus monthly DI of the WP. The results of the other 23 analyses are described in our supplement‐1 file. The layout and explanation of Figure 3 is very similar to those of wavelet analysis results of similar papers by us (Ehelepola & Ariyaratne, 2016; Ehelepola, Ariyaratne, & Dissanayake, 2021).

Figure 3's panel 3a illustrates the CWT of monthly Nino 4 SSTA. Panel 3a expands the time series into time frequency space. Figure 3's panel 3b shows a summary of the power for each period. The cross wavelet transform of the monthly Nino4 SSTA with monthly DI is illustrated by panel 3c. Figure 3's panel 3d shows a summary of the power for each period. Figure 3's panel 3e and 3f shows that wavelet coherence is highest between monthly DI and monthly Nino4 anomalies for yearly (period) cycles. The magnitudes of CWT, XWT, and WTC are illustrated by color‐coded panels on the right side of panels 3a, 3c and 3e. In those panels, dark blue and bright yellow indicate the lowest and highest magnitudes, respectively. The cone of influence is the thin U shaped black color lines in panels 3a, 3c and 3e. The 5% significance level of correlation between Nino4 index and DI using the red noise signal model is depicted by thick black color lines in panels 3a, 3c and 3e. The arrows in panels 3c and 3e are vectors demonstrating the phase difference. An arrow pointing vertically upward means the monthly DI lags behind the monthly Nino4 by 90°. A horizontal arrow pointing from left to right signifies two time series are in phase.

The wavelet coherence (WTC) results of the other analyses are given in our supplementary file.

Table 1 depicts the summary of correlations between teleconnection indices and DI of the WP as one and three districts of the province.

**Table 1:** The Summary of Wavelet Analysis Results‐ Teleconnections Versus Western Province Dengue

District	Nino 4	Nino3.4	EQSOI	MEI	DMI	EMI (Modoki)
Colombo District	DI peaks after 6.1 (0–9) months of Nino4 troughs	DI peaks after 3.4 (0–7) months of Nino3.4 peaks, DI peaks after 6.2 (0–10) months of Nino3.4 troughs	DI peaks after 3.2 months (0–5 months) of EQSOI troughs. DI peaks after 1.3 months (0–3 months) of EQSOI peaks	No correlation	DI peaks after 1.2 (0–3) months of DMI troughs	DI peaks after 2.4 months (1–4 months) of EMI peaks
Gampaha District	DI peaks after 1 month of Nino4 peaks DI peaks after 1.9 (1–2) months of Nino4 troughs	DI peaks after 2 (1–4) months of Nino3.4 peaks, DI peaks after 8.2 (1–13) months of Nino3.4 troughs	No correlation	DI peaks after 6.1 (1–9) months of MEI peaks, DI peak after 4.4 (1–7) months of MEI troughs	No correlation	No correlation
Kalutara District	DI peaks after 2.6 (0–5)months of Nino4 peaks	DI peaks after 4.7 (0–9) months of Nino3.4 peaks	DI peaks after 1 month (0–3 months) of EQSOI troughs	DI peaks after 4 (1–7) months of MEI peaks	DI peaks after 4 (1–7) months of DMI peaks, DI peaks after 9.8 (1–15) months of DMI troughs	No correlation
Western Province (As a whole)	DI peaks after about 1.6 months (0–2 months) of Nino 4 peaks DI peaks after about 4.6 months (varies 3–5 months) of Nino 4 troughs	DI peaks after about 1.6 months (0–2 months) of Nino3.4 peaks DI peaks after 4.7 months (3–6 months) of Nino 3.4 troughs	No correlation	DI peaks after 4.9 months (0.3–9.6 months) of EMI peaks DI peaks after 4.9 months (0. 3–9.8 months) of EMI troughs	No correlation	DI peaks after 4.9 months (0.3–9.6 months) of EMI peaks DI peak after 4.9 months (0. 3–9.8 months) of EMI troughs

Out of indices of ENSO, Nino 3.4 and Nino4 SSTA indices were correlated with DI of the WP and all three districts of the WP. MEI was correlated with DI of the WP and two districts. They have displayed a dual correlation pattern with DI of the WP and in some districts (both positive and negative extremes of teleconnection indices were followed by the rise of DI). EQSOI was correlated with DI of the two districts only and showed a dual correlation pattern in one of them. During the warm phase of ENSO, EQSOI is in the negative phase; the other three indices of ENSO are in the positive phase. Awareness of this is important to appreciate the results.

The DMI of the IOD did not show clear correlations with the DI of WP as a whole but correlated with the DI of two districts, showing a dual correlation pattern in one of them.

The EMI of ENSO Modoki displayed a dual correlation pattern with the DI of the WP but correlated with the DI of only one district.

## 4 Discussion

As showcased in Figures 2a–2d, DI of the WP and three districts of the WP showed a rising trend during 2005–2019 especially after 2009. In comparison DI rose 20‐fold during 2000–2012 and a further threefold from 2012 to 2019 in Sri Lanka (Malavige et al., 2021). In 2017 Sri Lanka had the worst ever recorded dengue epidemic to date (Tissera et al., 2020). That affected WP and all districts of the WP as illustrated by the DI peaks in Figures 2a–2d. According to one study, out of 25 districts of Sri Lanka, Colombo and Gampaha of the WP recorded the highest and second highest DI during that year (Tissera et al., 2020). However, those authors concluded that climatic factors other than rainfall did not explain the rise of DI in 2017 in Sri Lanka (Tissera et al., 2020).

Table 2 summarizes the interpretation of our results.

**Table 2:** The Summary of Interpretation of Our Results

	El Nino Southern oscillation (ENSO)	Indian ocean dipole (IOD)	ENSO Modoki
Western Province (As a whole)	Both positive and negative extremes of ENSO result rise of DI	No influence on DI detected	Both positive and negative extremes of ENSO Modoki result rise of DI
Colombo District	Both positive and negative extremes of ENSO result rise of DI	Negative extremes of IOD results rise of DI	Positive extremes of IOD results rise of DI
Gampaha District	Both positive and negative extremes of ENSO result rise of DI	No influence on DI detected	No influence on DI detected
Kalutara District	Positive extremes of ENSO results rise of DI	Both positive and negative extremes of IOD result rise of DI	No influence on DI detected

In the introduction, we have described in detail how weather parameters modulate DI, how teleconnections modulate weather parameters in the WP and the reliability of our method of analysis. Therefore, we can reliably infer that teleconnections modulate the DI of the WP, as depicted in Table 2. The following reasons explain the differences of results of the province and its districts. DI of a locality is the net effect of several weather and many other factors (Abdulla et al., 2002; Ebi & Nealon, 2016). There are local variations of those factors. Teleconnections modulate the rainfall (weather) of different weather stations in WP differently, as we describe below (Abeysekera et al., 2021). We used administrative boundaries to demarcate districts. Weather and other factors that modulate DI do not adhere to those boundaries. Only one index of IOD and ENSO Modoki was available for our study. For example, had we analyzed the correlation between the DI of Gamapha district and the IOD using two indices of the IOD, we might have found a correlation with one of the indices.

Our SSTA results indicate that DI of the WP as a whole and DI of two districts of the WP get raised by extremes of both phases of ENSO (El Ninos as well as La Ninas), a dual correlation pattern. The detection of broadly similar dual correlation patterns in two districts individually and in WP as a whole enhances the fidelity of our results. When we consider ENSO, in Colombo district, the results of Nino3.4, Nino4 and EQSOI are similar but there is no correlation with MEI (Table 1). In Gampaha district results Nino3.4, Nino4 and MEI are similar but there is no correlation with EQSOI. In Kalutara district, only positive phase extremes of ENSO were followed by the rise of DI. It is important to note that different indices of ENSO never showed opposite correlation patterns (Table 1). When they show a single correlation that agrees with one of the dual correlation patterns of the WP and other district(s). However, DI was not correlated with EQSOI and MEI in some districts. The conclusions of past studies on this topic from South Asia are based on the results of one SSTA index only (Atique et al., 2016; Banu et al., 2015; Kakarla et al., 2019; Pramanik et al., 2020). Thus, we think our results may be more reliable.

Rainfall was demonstrated to be correlated with the DI of each district of WP by past studies (Prabodanie et al., 2020; Tissera et al., 2020; Withanage et al., 2018). Two correlation patterns between ENSO and rainfall in Sri Lanka were demonstrated by past studies (Abeysekera et al., 2021; Hapuarachchi & Jayawardene, 2018; Zubair et al., 2003). El Ninos (La Ninas) reduce (increase) the rainfall in Sri Lanka during the First Inter Monsoon (April) and South West Monsoon = Indian summer monsoon (May‐September) and North East Monsoon = Indian winter monsoon (December–February) (Abeysekera et al., 2021; Burt & Weerasinghe, 2014; Hapuarachchi & Jayawardene, 2018; Zubair et al., 2003). During El Ninos, rainfall increases from October–December, and decreases from January–March (Abeysekera et al., 2021; Hapuarachchi & Jayawardene, 2018). During La Ninas throughout the Second Inter Monsoon Period (mainly October) rainfall decreased (Abeysekera et al., 2021). Those past findings help us understand the dual correlation pattern we observed.

Some authorities suggest a pressure anomaly index (EQSOI) might be more suitable for Southern Asia (Sri Lanka) to study ENSO related health outcomes (McGregor & Ebi, 2018). Nevertheless, our results show SSTA are better correlated with the DI of WP in Sri Lanka. Getting results that are broadly in agreement using two SSTA indices, one atmospheric change index (EQSOI) and one blended index (MEI), enhances the trustworthiness of our results. The availability of only one index of IOD and ENSO Modoki restricted us from doing analysis with multiple indices and getting a better idea.

Negative phase extremes of IOD were followed by the rise of DI in Colombo district (Table 1). In Kalutara district, extremes of both phases of IOD were followed by an increase in DI (dual correlation). No correlation was seen in Gampaha district or in the WP. One past study showed that indices of ENSO are better correlated with rainfall in some weather stations in Sri Lanka than the index of IOD (Burt & Weerasinghe, 2014). Positive IODs enhance rainfall during September to December but not so during January to May in Sri Lanka (Burt & Weerasinghe, 2014; Zubair et al., 2003). An increase in rainfall in October‐November during the IOD positive years and a decrease in IOD negative years were revealed by a recent study from Sri Lanka (Abeysekera et al., 2019). Therefore, the rise of DI following the negative phase of IOD may not be related to rainfall but may be via by another weather parameter like temperature. Leptospirosis is another weather sensitive emerging infectious disease. Rainfall, temperature and humidity ranges that favor dengue transmission favor leptospirosis transmission as well (Ebi & Nealon, 2016; Ehelepola, Ariyaratne, & Dissanayake, 2021; Ehelepola et al., 2015). Therefore, we mention the results of two related studies for comparison. Extremes of both positives and negatives of IOD resulted in rise of leptospirosis incidence of Hambantota districts of Sri Lanka (Ehelepola, Ariyaratne, & Dissanayake, 2021). Nevertheless, only the positive extremes of IOD resulted rise of leptospirosis incidence in Kandy district (Ehelepola, Ariyaratne, Aththanayake, et al., 2021).

Both extremes of EMI (El Nino Modoki and La Nina Modoki) were followed by the rise in DI of the WP as a whole (Table 1). Positive extremes were followed by a rise in DI only in Colombo district. Negative extremes were not correlated with the DI of any individual district. A past study has demonstrated that strong La Nina Modoki conditions ensuing higher rainfall in January and February and below normal rainfall in May and October, while strong El Nino Modoki events causing lower rainfall during months of February and July and above normal rainfall during months of August, September and October in Sri Lanka (Hapuarachchi & Jayawardene, 2018). Those findings are useful to understand the existence of two correlation patterns between EMI and DI.

Teleconnections do not modulate the weather parameters of different weather stations in WP in a uniform manner (Abeysekera et al., 2021). For example, the numbers of wet days were significantly reduced by negative IODs in Colombo and Ratmalana but not in Katunayake and Gampaha‐Henarathgoda weather stations, during the second inter‐monsoon period (Abeysekera et al., 2021). The number of wet days was positively correlated with DI in Sri Lanka (Ehelepola et al., 2015). Most of the Colombo population has access to pipe‐borne water, so they do not store water indoors during dry periods. Hence, dengue transmission in WP during relatively dry periods is not mainly due to *Aedes* breeding indoors. These facts further support our aforesaid conclusion that the rise of DI following the negative phase of IOD may not be related to rain.

The average lag periods we observed vary from district to district, and even in the same district there is a range. Teleconnections do not modulate the weather in WP uniformly, as described. The awareness that the intensity, area affected by and duration of each ENSO and other teleconnection event are unique is also helpful to appreciate these variations (McGregor & Ebi, 2018). Weather is only one key factor influencing dengue transmission. Dengue incidence of an area is the net effect of several factors (Ebi & Nealon, 2016; Ehelepola et al., 2015; Liyanage et al., 2016; World Health Organization, 2024). Therefore, the relative importance of weather in determining the DI of the locality varies from area to area and even in the same area with time (Ebi & Nealon, 2016; Ehelepola et al., 2015; Liyanage et al., 2016). That knowledge helps to understand the variations of results in three districts and the diversity of lag periods as well. When considering the life cycles of the vector and the virus, lag periods we observed are plausible. Lag periods ranging from 1 to 12 months were reported by past similar studies (Atique et al., 2016; Banu et al., 2015; Hu et al., 2010; Pramanik et al., 2020; Tipayamongkholgul et al., 2009).

### 4.1 Comparison With Past Studies

The only published study from Sri Lanka on teleconnection dengue correlation was conducted in Kalutara district. It demonstrated that only El Ninos increase temperature, rainfall and DI in that chronological order (Liyanage et al., 2016). Those authors used one SSTA index (Oceanic Nino Index = ONI). We using different indices of ENSO and a different method for analysis have confirmed their findings. There was only one strong El Nino during that 5 year study (Liyanage et al., 2016). In comparison, in our 15 years study we considered not only El Nino/La Ninas but all positive and negative phases of ENSO, which helped us detect the dual correlation. For example, in Gampaha, our detection of rise of DI following positive phase extremes of ENSO was based on 26/83 peaks of Nino4 index and rise of DI following negative phase extremes of ENSO was based on 31/83 troughs of Nino4 index. There is a meeting abstract on the interrelationship between DI of Sri Lanka and ENSO and IOD (Nijamdeen et al., 2020). Details of their methodology and findings are not publicly available. Those authors found that the worst recorded dengue epidemic in Sri Lanka in 2017 was preceded by high temperatures due to IOD. Another study was done in Kalutara district using the ONI index of the ENSO (Liyanage et al., 2022). It demonstrated that following El Ninos, *Aedes* became more abundant, which is helpful for dengue transmission. That further supports our results and the results of Tissera et al., 2020. In India (Sri Lanka's closest neighbor) following positive extremes of ENSO dengue case count rose in some states while in some other states it declined (Pramanik et al., 2020). This is different from the dual correlation we identified in WP, as positive extremes of ENSO never decreased DI in WP. Those authors used ONI for the study. Another seven year study from India demonstrated positive extremes of Nino3.4 and DMI indices (respectively in 2015 and 2016) followed by an increase of weekly dengue cases (Kakarla et al., 2019). There were cross‐correlations between Nino3.4 and DMI indices with dengue cases. Negative extremes of ENSO (Nino 3.4) were associated with the rise of reported dengue cases in Lahore District of Pakistan in 2006–2014 period (Atique et al., 2016). IOD demonstrated a synchronized pattern with the 2010–2012 dengue peak in that study (Atique et al., 2016). ENSO (Nino 3.4) and IOD (DMI) were correlated with DI in Bangladesh, and this was the first time series analysis that showed a correlation between IOD and DI (Banu et al., 2015). This correlation might occur via the association of DI between temperature and rainfall, the authors concluded (Banu et al., 2015). Those are similar past studies from South Asian countries. The results of those (except part of the results of Pramanik et al., 2020 agree with ours. All past published studies from South Asia have used a single SSTA index of ENSO, either Nino 3.4 or ONI. There are several other ENSO dengue correlation studies from the rest of the Asia Pacific, Australia and Americas illustrating how ENSO modulates DI (Anyamba et al., 2019; Chuang et al., 2017; García et al., 2023; Hu et al., 2010; McGregor & Ebi, 2018; Naish et al., 2014; Tipayamongkholgul et al., 2009; Yip et al., 2022). Out of those, a study from Taiwan (in the Pacific) has demonstrated both ENSO and IOD might be contributing to unusual dengue outbreaks by modulating local weather and IOD may be more relevant to the inter‐annual epidemic pattern (Chuang et al., 2017). We found that on an island in the Indian Ocean ENSO has a more uniform correlation with DI than IOD. Interestingly, those authors have evaluated MEI and all Nino indices of ENSO and found Nino 3.4 had high coherence with major dengue outbreaks (Chuang et al., 2017). A study from New Caledonia using data of 40 years detected significant inter‐annual correlations between ENSO and local weather parameters but not between ENSO and dengue (Descloux et al., 2012). A study from Singapore shows that temperature, relative humidity and SOI of ENSO (but not rainfall) were independently associated with dengue case count (Earnest et al., 2012). SOI's independent association of their results is interesting. Malaria is another mosquito‐borne weather sensitive disease. Only one past study on teleconnection disease correlation has considered all three teleconnections we considered in this study (Imai et al., 2016). Those researchers found generally a negative correlation between malaria cases in Papua New Guinea and Nino 3.4, EMI, and DMI (Imai et al., 2016). Although no past time series analysis has demonstrated a correlation between both extremes of ENSO and DI like us, a study from Colombia has elicited such a correlation between ENSO and leptospirosis (Arias‐Monsalve & Builes‐Jaramillo, 2019).

### 4.2 Limitations

Reported dengue case data was based on standard national surveillance case definitions based on 1997/2011 WHO criteria (Liyanage et al., 2016). Some notified dengue cases, especially during the first decade of our study period, were not serologically confirmed. Reported cases here are usually hospitalized dengue cases and only the tip of the dengue iceberg (Ehelepola et al., 2015; Liyanage et al., 2016). Asymptomatic seroconversions, mild dengue fever cases (do not seek care) and even some hospitalized cases are not reported as mild cases of many other legally notifiable infections. Intern doctors in the hospitals usually do the reporting. During the last decade, infection control nurses have also started to support the notification process. Periodic reminders to them and private sector doctors may increase reporting. Unmeasured confounders could have affected the results; such confounders include, but are not limited to, arrival of new genotypes of dengue virus in the population, behaviors and movements of population, changes in population density, the effects of preventive work and changes in herd immunity (Ebi & Nealon, 2016; Ehelepola et al., 2015; Tissera et al., 2020; World Health Organization, 2024).

## 5 Conclusions

We conclude that both positive and negative phase extremes of ENSO, IOD and ENSO Modoki increase the DI of WP via modulation of local weather. However, those teleconnections modulate the DI of different districts of the WP in different ways. This knowledge can be utilized to increase the efficiency of dengue control work (Thomson et al., 2018). Extreme ENSO and extreme IOD events are likely to occur more frequently with the on‐going climate changes (Johnson et al., 2020; Wang et al., 2019). That may lead to creating favorable weather conditions for dengue transmission in the WP and elsewhere more frequently.

Information on the extremes of teleconnection indices can be easily obtained from the sources mentioned in our data statement. Preventive health authorities in Sri Lanka can regularly monitor those sources, detect extremes of teleconnection indices, escalate integrated dengue preventive work during the lag periods and prevent/blunt impending dengue peaks and prepare to cater to more dengue patients. Sri Lanka is undergoing the worst economic crisis since gaining independence that reached its zenith in mid‐2022. Thus, finding resources for additional dengue preventive work will be difficult. The government's reduction of recruitment of HCW with emigrations to escape economic woes resulted in a decline in available health manpower. A study done in WP illustrates that personnel costs accounted for about 89% of the total dengue prevention cost and how civil‐military cooperation dengue vector control programs reap public health improvement while being cost‐effective (Liyanage et al., 2019). We recommend escalating such programs and public education on vector control and mosquito bite prevention after noticing the extremes of teleconnections. Within existing budget limits, initiating a social media based program based on behavioral sciences would be useful (World Health Organization, 2021). We think this method can be applied elsewhere after determining the correlation pattern between teleconnection indices and DI in those localities. ENSO was proposed as an early warning tool for dengue outbreak in India and in many other tropical countries lately (McGregor & Ebi, 2018; Pramanik et al., 2020). However, there is a caveat. No two ENSO/teleconnection events are the same (McGregor & Ebi, 2018). Hence, we shall not expect consistent temporal or spatial teleconnection‐dengue associations and lose our trust in this correlation just because DI does not rise after one extreme of a teleconnection in one district. The awareness of teleconnections is low among most Sri Lankan public health workers and policymakers we know. More publications like this may contribute to improving that situation to some extent.

## Conflict of Interest

The authors declare no conflicts of interest relevant to this study.

**List of abbreviations**
ENSO: El Nino Southern Oscillation
IOD: Indian Ocean Dipole
DI: Dengue incidence
WP: Western Province
EQSOI: Equatorial Southern Oscillation Index
MEI: Multivariate ENSO Index
DMI: Dipole mode index
EMI: El Nino Modoki Index
SST: Sea surface temperature
SSTA: Sea surface temperature anomaly
SOI: Southern Oscillation Index
USA: United States of America
CWT: Continuous wavelet transform
XWT: Cross wavelet transform
WTC: Wavelet coherence

## Supporting information

Supporting Information S1

## Data Availability

We have used secondary data and we do not own them. We have obtained the reported case numbers of dengue in Western Province from the printed weekly epidemiology reports of the Ministry of Health of Sri Lanka 2005–2019. It is published and available at the Epidemiology Unit, Ministry of Health of Sri Lanka, 231 De Saram Place, Colombo 10, Sri Lanka. Also available at the library of the Faculty of Medicine, University of Peradeniya, Sri Lanka and some other medical libraries of Sri Lanka. Population data can be obtained from Sri Lanka Department of Census and Statistics, “Sankayana Mandiraya” No. 306/71, Polduwa Road, Battaramulla, Sri Lanka. Part of that data is available online as well: http://www.statistics.gov.lk/Resource/en/Population/Vital_Statistics/Mid-year_population_by_district.pdf (updated URL: https://www.statistics.gov.lk/Resource/en/Population/Vital_Statistics/Mid-year_population_by_district_and_sex_2024.pdf). Monthly Nino 3.4 and Nino 4 and SOI indices, plus monthly DMI data is available online from the NOAA Physical Sciences Laboratory, National Oceanic and Atmospheric Administration of the United States: https://www.psl.noaa.gov/gcos_wgsp/Timeseries/ (updated URL: https://www.psl.noaa.gov/data/timeseries/month/). Monthly EMI data was obtained from Dr. Takeshi Doi of Japan Agency for Marine‐Earth Science and Technology (JAMSTEC). JAMSTEC Monthly El Nino Modoki Index data https://www.jamstec.go.jp/aplinfo/sintexf/e/elnmodoki/data.html (updated URL: https://www.jamstec.go.jp/virtualearth/general/en/index.html).
