# Supplementary material for: The Correlation Between Three Teleconnections and Dengue Incidence in the Western Province of Sri Lanka, 2005–2019
Source: Geohealth. 2025 Sep 23;9(9):e2024GH001144. doi: 10.1029/2024GH001144 (PMC12455246; doi:10.1029/2024GH001144)
Supplement: Supplementary file 1 — Supporting Information S1 [file GH2-9-e2024GH001144-s001.pdf]

AUG GeoHealth Journal

Supporting Information for

The Correlation Between Three Teleconnections and Dengue Incidence in the Western Province of Sri Lanka, 2005-2019.

Authors: N.D.B.Ehelepola[1]*, Kusalika Ariyaratne[2], R.M.P.Ratnayake[3]

Affiliations:

[1]Affiliation for author 1. Teaching (General) Hospital-Peradeniya, Peradeniya, Sri Lanka.

[2]Affiliation for author 2. Lanka Hydraulic Institute, Moratuwa, Sri Lanka.

[3]Affiliation for author 3. National Hospital-Kandy, Kandy, Sri Lanka.

Contents of this file:

Results of the wavelet analysis of monthly teleconnection indices vs. monthly DI of the studied area are shown here. There are 24 figures (Colombo District 1-6, Gampaha District 7-12, Kalutara District 13-18 and the Western Province 19-24). The table at the end summarizes the results of these 24 analyses.

Figure 1: Wavelet analysis results of monthly Nino 4 SSTA vs. dengue incidence of Colombo District for 2004-2019

Continuous Wavelet Transform (CWT) of monthly NINO4 SSTA Left Panel (1a): CWT, Right Panel (1b): Wavelet power

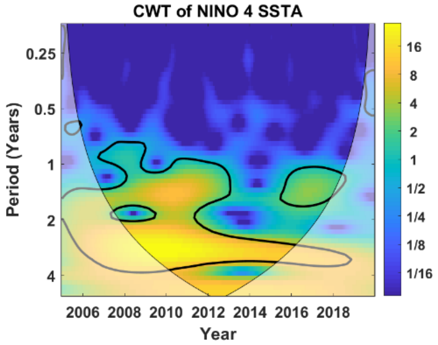

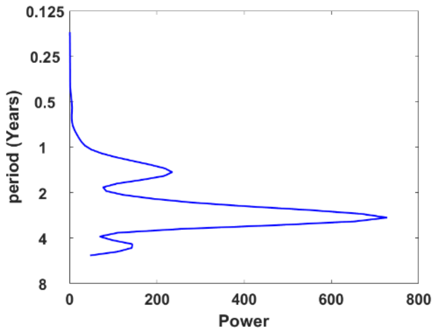

Cross Wavelet Transform (XWT) Left Panel (1c): XWT, Right Panel (1d): Wavelet

power of XWT

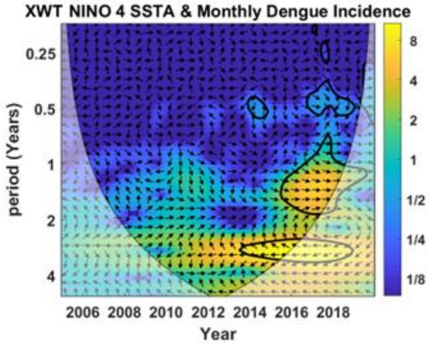

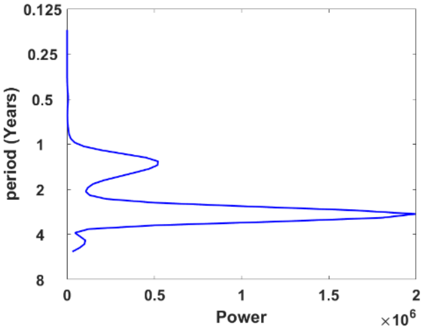

Cross Coherence (WTC) Left Panel (1e): WTC, Right Panel (1f): Wavelet power of WTC

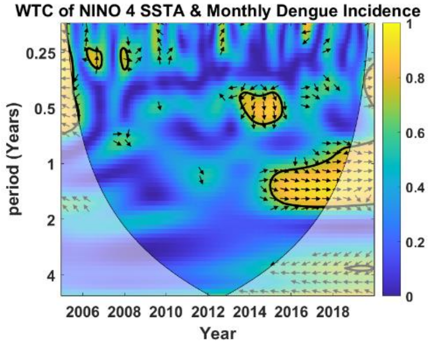

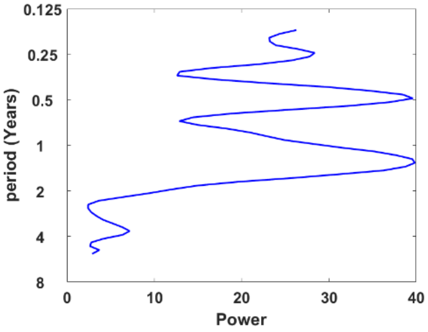

Reconstructed time series for selected period(1g)

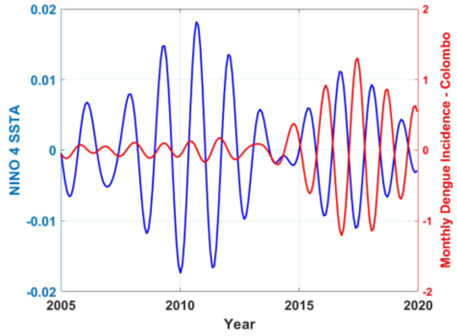

Figure 1: Wavelet analysis results of monthly Nino 4 SSTA vs. dengue incidence of Colombo District for 2004-2019: Panel 1a- continuous wavelet transform (CWT) variations; Panel 1b- wavelet power of CWT; Panel 1c- cross wavelet transform (XWT) variations; Panel 1d- wavelet power of XWT; Panel 1e- wavelet coherence (WTC); Panel 1f- wavelet power of WTC; Panel 1g- reconstructed time series for 2005-2019.

Figure 2: Wavelet analysis results of monthly Nino 3.4 SSTA vs. dengue incidence of Colombo District for 2004-2019

Continuous Wavelet Transform (CWT) of monthly NINO3.4 SSTA Left Panel (2a): CWT, Right Panel (2b): Wavelet power

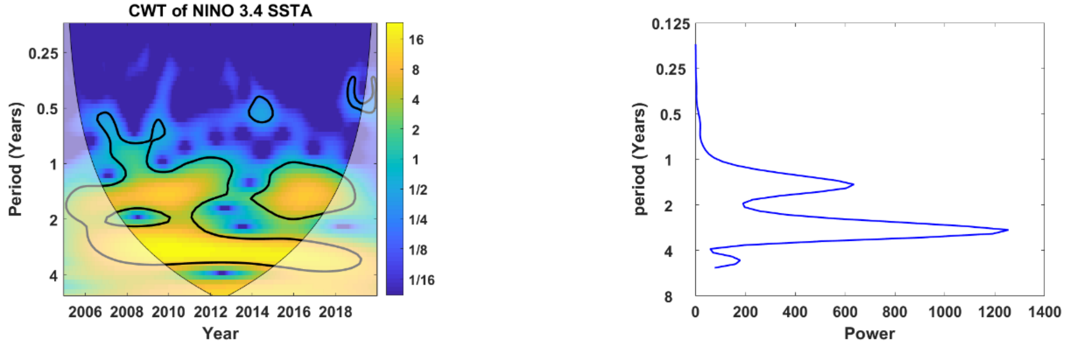

Cross Wavelet Transform (XWT) Left Panel (2c): XWT, Right Panel (2d): Wavelet power of XWT

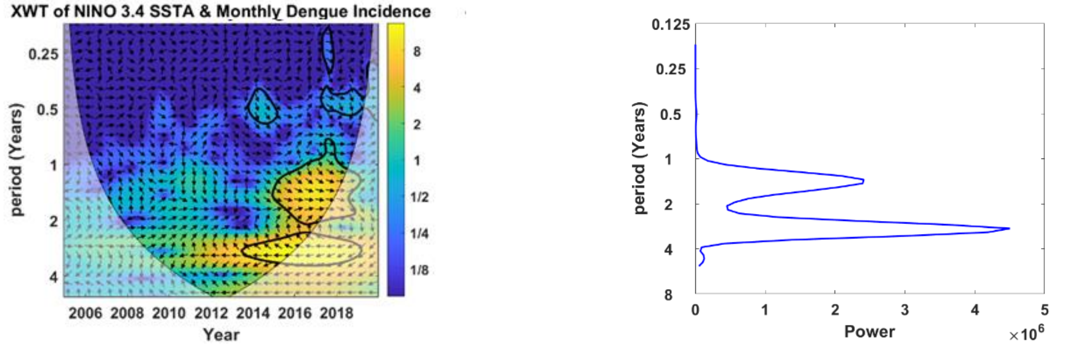

Cross Coherence (WTC) Left Panel (2e): WTC, Right Panel (2f): Wavelet power of WTC

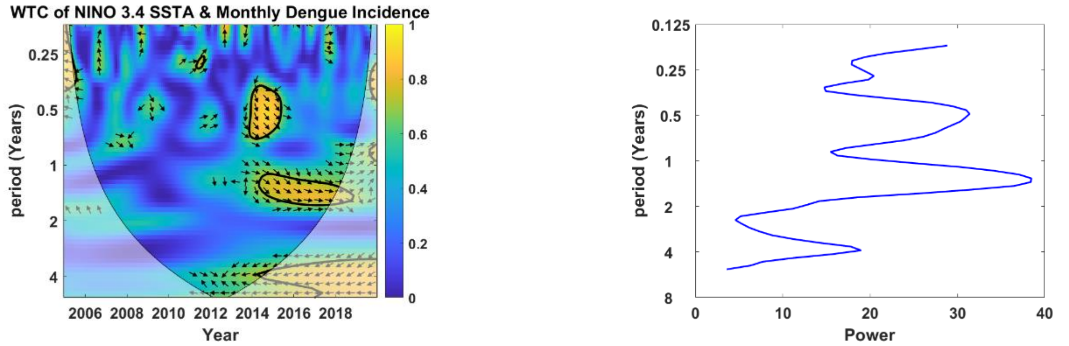

Reconstructed time series for selected period (2g)

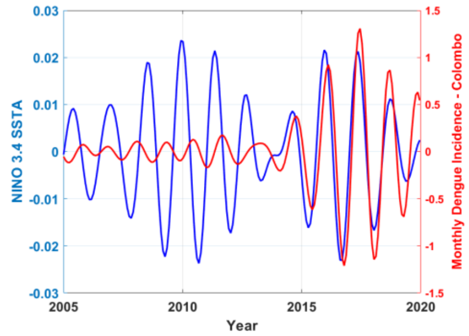

Figure 2: Wavelet analysis results of monthly Nino 3.4 SSTA vs. dengue incidence of Colombo District for 2004-2019: Panel 2a- continuous wavelet transform (CWT) variations; Panel 2b- wavelet power of CWT; Panel 2c- cross wavelet transform (XWT) variations; Panel 2d- wavelet power of XWT; Panel 2e- wavelet coherence (WTC); Panel 2f- wavelet power of WTC; Panel 2g- reconstructed time series for 2005-2019.

Figure 3: Wavelet analysis results of monthly EQSOI vs. dengue incidence of Colombo District for 2004-2019

Continuous Wavelet Transform (CWT) of monthly EQSOI Left Panel (3a): CWT, Right Panel (3b): Wavelet power

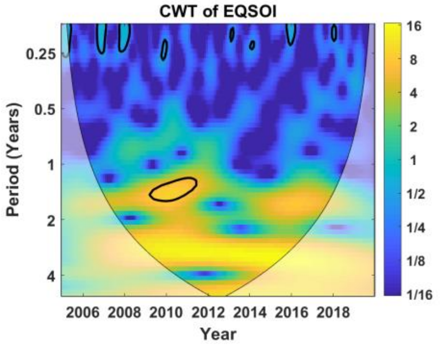

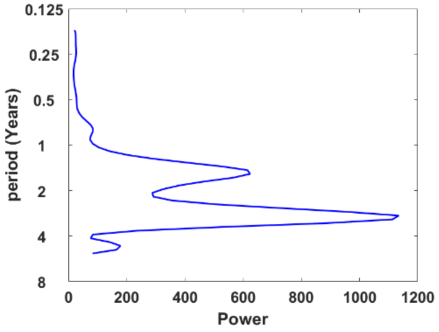

Cross Wavelet Transform (XWT) Left Panel (3c): XWT, Right Panel (3d): Wavelet power of XWT

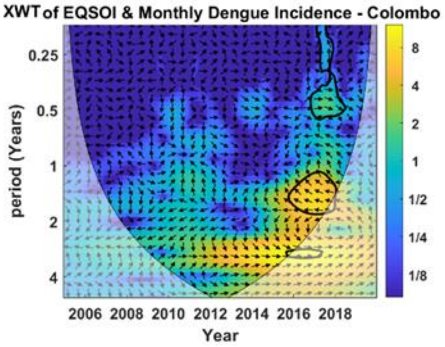

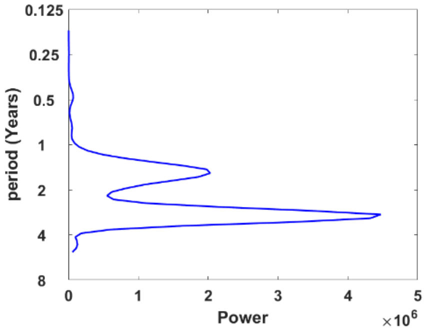

Cross Coherence (WTC) Left Panel (3ce): WTC, Right Panel (3f): Wavelet power of WTC

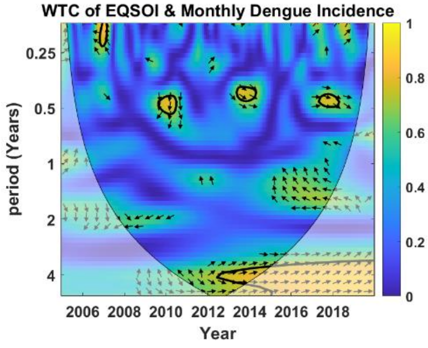

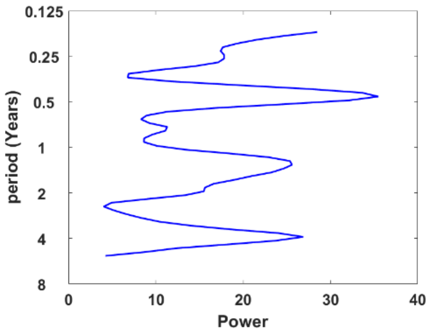

Reconstructed time series for selected period

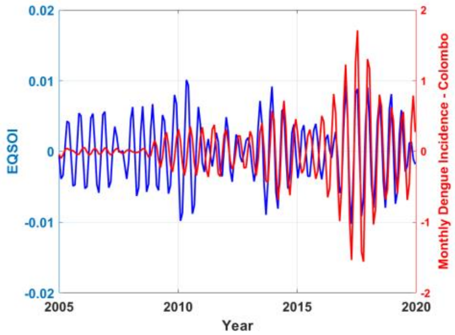

Figure 3: Wavelet analysis results of monthly EQSOI vs. dengue incidence of Colombo District for 2004-2019: Panel 3a- continuous wavelet transform (CWT) variations; Panel 3b- wavelet power of CWT; Panel 3c- cross wavelet transform (XWT) variations; Panel 3d- wavelet power of XWT; Panel 3e- wavelet coherence (WTC); Panel 3f- wavelet power of WTC; Panel 3g- reconstructed time series for 2005-2019.

Figure 4: Wavelet analysis results of monthly MEI vs. dengue incidence of Colombo District for 2004-2019.

Continuous Wavelet Transform (CWT) of monthly MEI Left Panel (4a): CWT, Right Panel (4b): Wavelet power

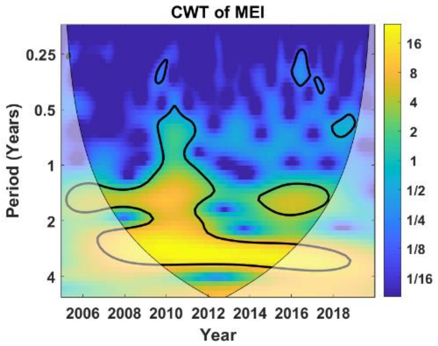

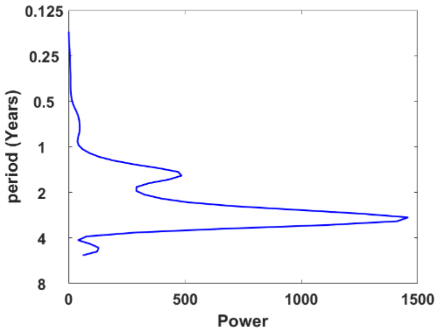

Cross Wavelet Transform (XWT) Left Panel (4c): XWT, Right Panel (4d): Wavelet power of XWT

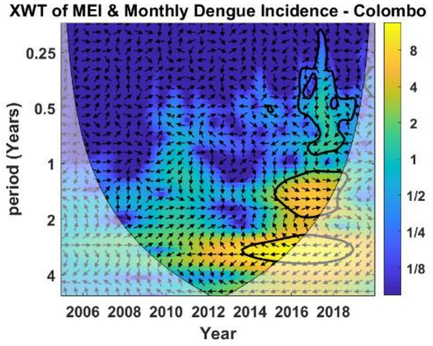

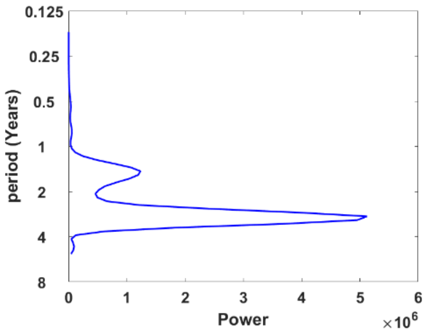

Cross Coherence (WTC) Left Panel (3ce): WTC, Right Panel (4f): Wavelet power of

WTC

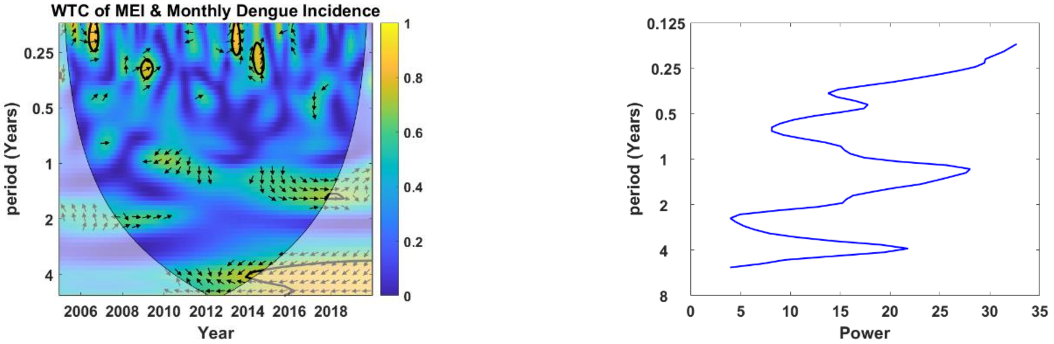

Reconstructed time series for selected period (4g)

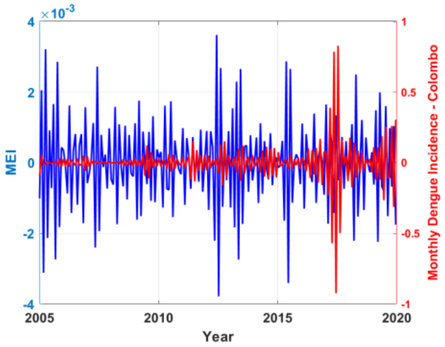

Figure 4: Wavelet analysis results of monthly MEI vs. dengue incidence of Colombo District for 2004-2019: Panel 4a- continuous wavelet transform (CWT) variations; Panel 4b- wavelet power of CWT; Panel 4c- cross wavelet transform (XWT) variations; Panel 4d- wavelet power of XWT; Panel 4e- wavelet coherence (WTC); Panel 5f- wavelet power of WTC; Panel 4g- reconstructed time series for 2005-2019.

Figure 5: Wavelet analysis results of monthly DMI vs. dengue incidence of Colombo District for 2004-2019

Continuous Wavelet Transform (CWT) of monthly DMI Left Panel (5a): CWT, Right Panel (5b): Wavelet power

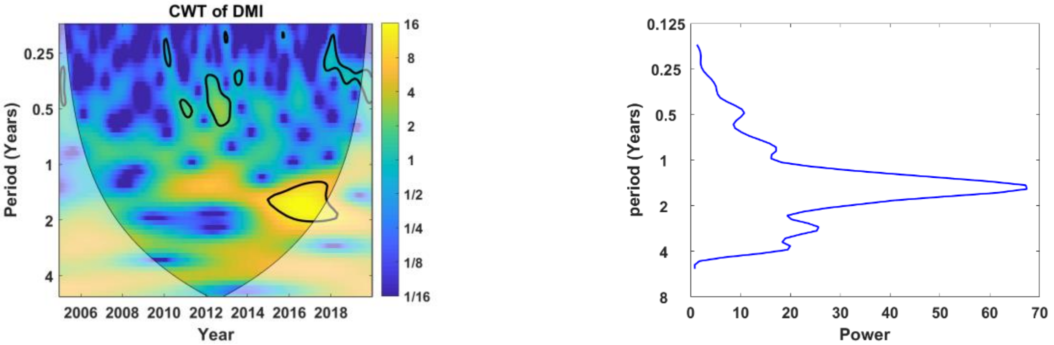

Cross Wavelet Transform (XWT) Left Panel (5c): XWT, Right Panel (5d): Wavelet

power of XWT

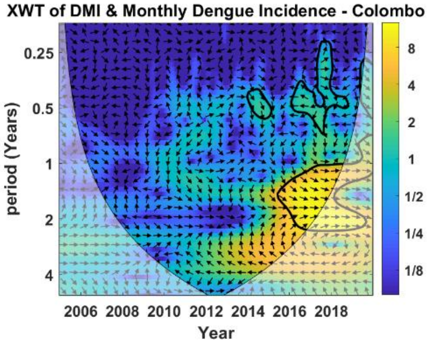

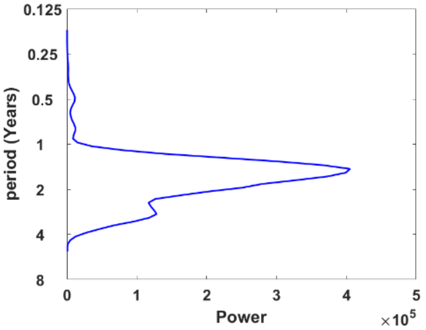

Cross Coherence (WTC) Left Panel (3ce): WTC, Right Panel (5f): Wavelet power of WTC

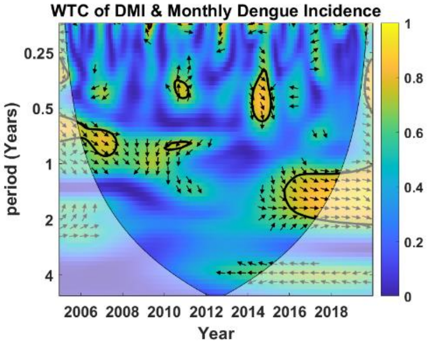

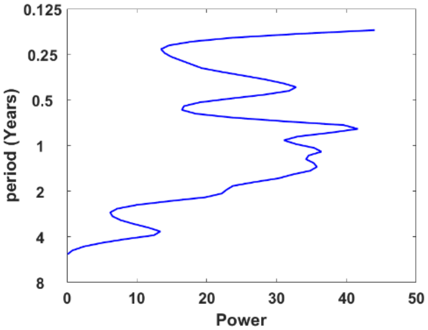

Reconstructed time series for selected period (5g)

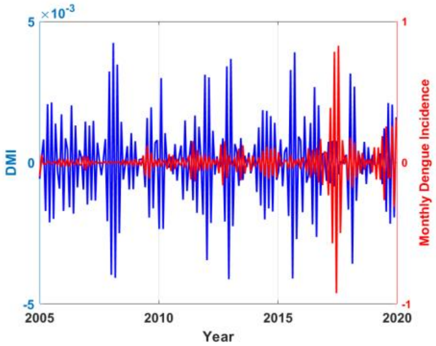

Figure 5: Wavelet analysis results of monthly DMI vs. dengue incidence of Colombo District for 2004-2019: Panel 5a- continuous wavelet transform (CWT) variations; Panel 5b- wavelet power of CWT; Panel 5c- cross wavelet transform (XWT) variations; Panel 5d- wavelet power of XWT; Panel 5e- wavelet coherence (WTC); Panel 5f- wavelet power of WTC; Panel 5g- reconstructed time series for 2005-2019.

Figure 6: Wavelet analysis results of monthly EMI vs. dengue incidence for of Colombo District for 2004-2019.

Continuous Wavelet Transform (CWT) of monthly EMI Left Panel (6a): CWT, Right

Panel (6b): Wavelet power

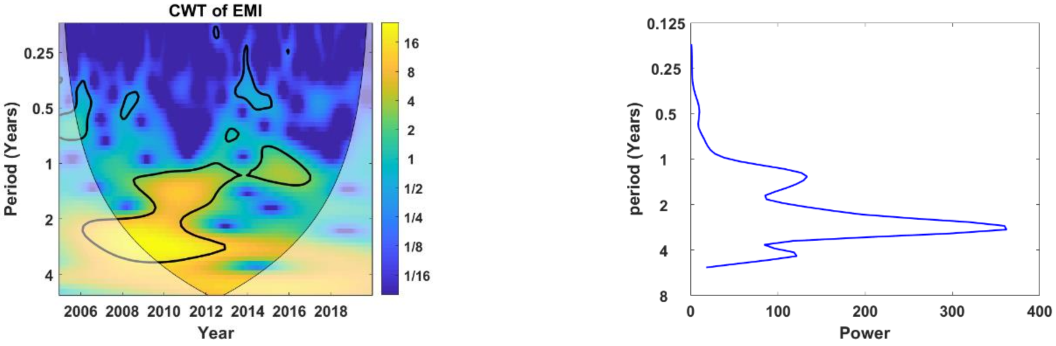

Cross Wavelet Transform (XWT) Left Panel (6c): XWT, Right Panel (6d): Wavelet power of XWT

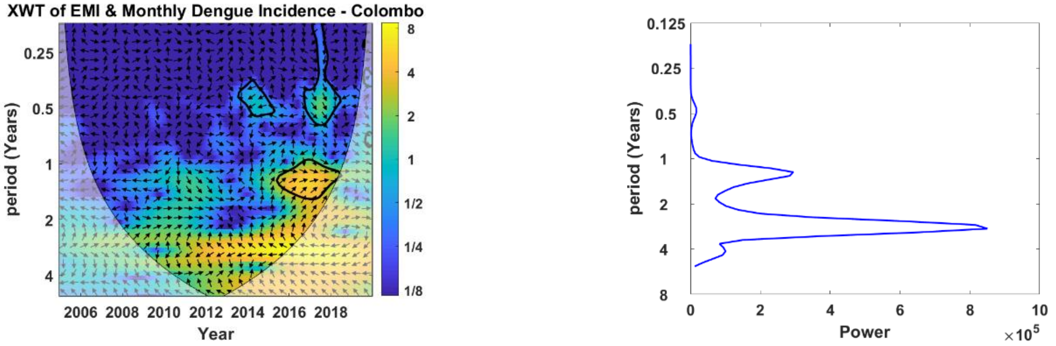

Cross Coherence (WTC) Left Panel (6e): WTC, Right Panel (6f): Wavelet power of WTC

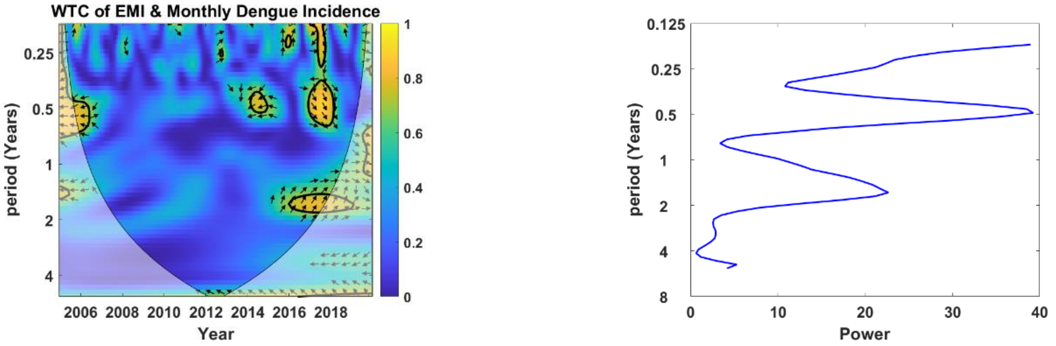

Reconstructed time series for selected period (6g)

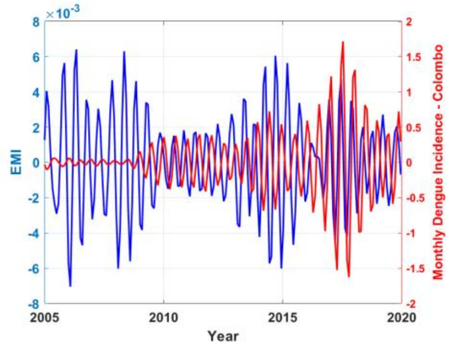

Figure 6: Wavelet analysis results of monthly EMI vs. dengue incidence for of Colombo District for 2004-2019: Panel 6a- continuous wavelet transform (CWT) variations; Panel 6b- wavelet

power of CWT; Panel 6c- cross wavelet transform (XWT) variations; Panel 6d- wavelet power of XWT; Panel 6e- wavelet coherence (WTC); Panel 6f- wavelet power of WTC; Panel 6g- reconstructed time series for 2005-2019.

……………………………………………………………………………………..

Figure 7: Wavelet analysis results of monthly Nino 4 SSTA vs. dengue incidence of Gampaha District for 2004-2019.

Continuous Wavelet Transform (CWT) of monthly NINO4 SSTA Left Panel (7a): CWT, Right Panel (7b): Wavelet power

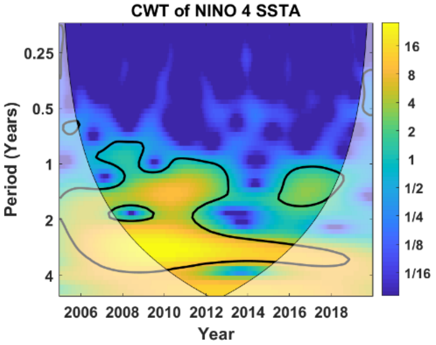

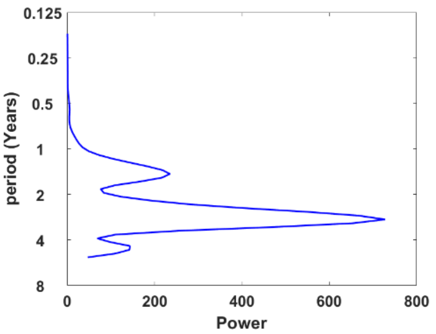

Cross Wavelet Transform (XWT) Left Panel (7c): XWT, Right Panel (7d): Wavelet power of XWT

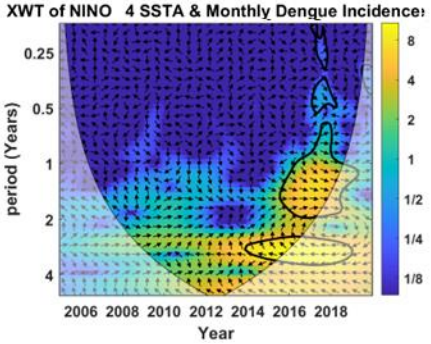

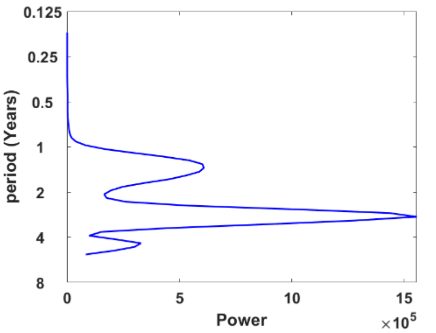

Cross Coherence (WTC) Left Panel (7e): WTC, Right Panel (7f): Wavelet power of WTC

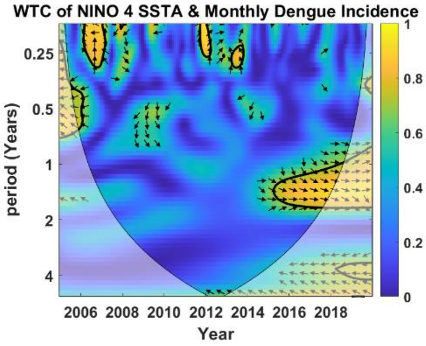

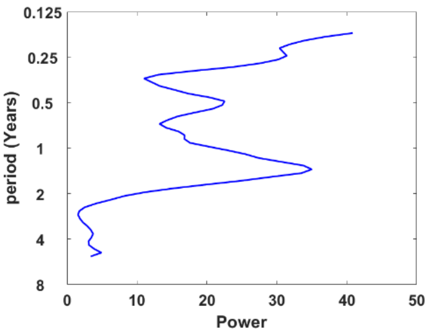

Reconstructed time series for selected period (7f)

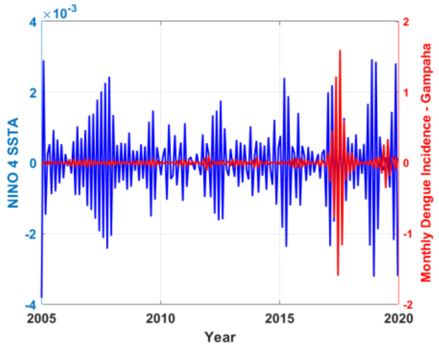

Figure 7: Wavelet analysis results of monthly Nino 4 SSTA vs. dengue incidence of Gampaha District for 2004-2019: Panel 7a- continuous wavelet transform (CWT) variations; Panel 7b- wavelet power of CWT; Panel 7c- cross wavelet transform (XWT) variations; Panel 7d- wavelet power of XWT; Panel 7e- wavelet coherence (WTC); Panel 7f- wavelet power of WTC; Panel 7g- reconstructed time series for 2005-2019.

Figure 8: Wavelet analysis results of monthly Nino 3.4 SSTA vs. dengue incidence of Gampaha District for 2004-2019.

Continuous Wavelet Transform (CWT) of monthly NINO3.4 SSTA Left Panel (8a): CWT, Right Panel (8b): Wavelet power

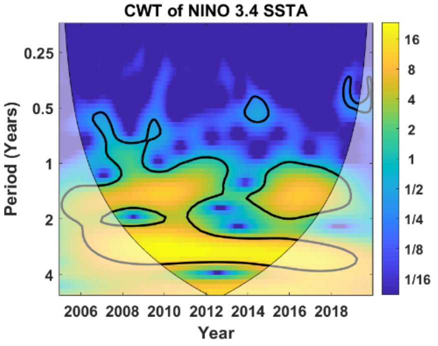

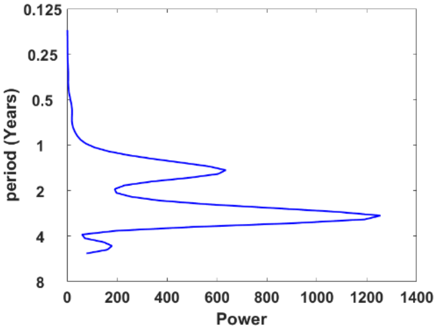

Cross Wavelet Transform (XWT) Left Panel (8c): XWT, Right Panel (8d): Wavelet power of XWT

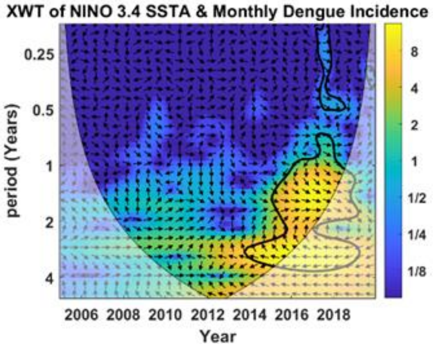

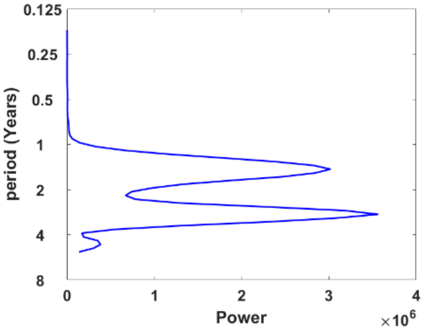

Cross Coherence (WTC) Left Panel (8e): WTC, Right Panel (8f): Wavelet power of WTC

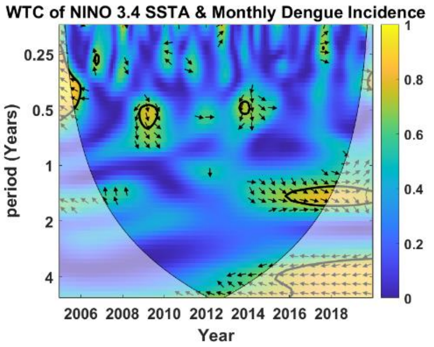

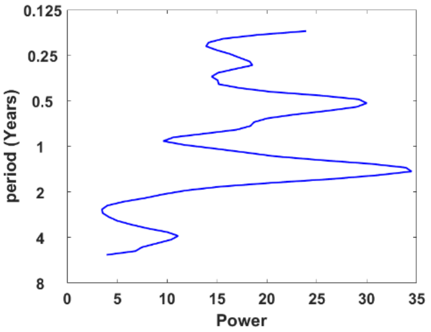

Reconstructed time series for selected period (8g)

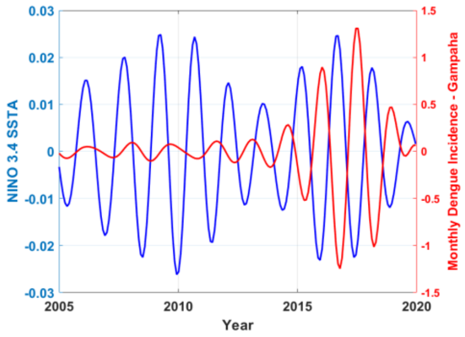

Figure 8: Wavelet analysis results of monthly Nino 3.4 vs. dengue incidence of Gampaha District for 2004-2019: Panel 8a- continuous wavelet transform (CWT) variations; Panel 8b- wavelet power of CWT; Panel 8c- cross wavelet transform (XWT) variations; Panel 8d- wavelet power of XWT; Panel 8e- wavelet coherence (WTC); Panel 8f- wavelet power of WTC; Panel 8g- reconstructed time series for 2005-2019.

Figure 9: Wavelet analysis results of monthly EQSOI vs. dengue incidence of Gampaha District for 2004-2019.

Continuous Wavelet Transform (CWT) of monthly EQSOI Left Panel (9a): CWT, Right Panel (9b): Wavelet power

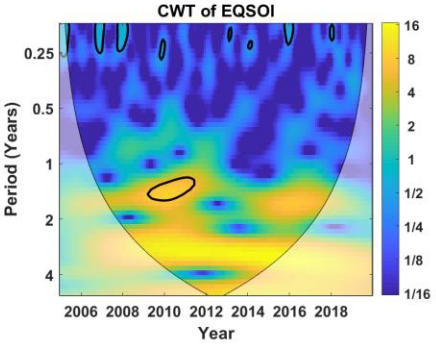

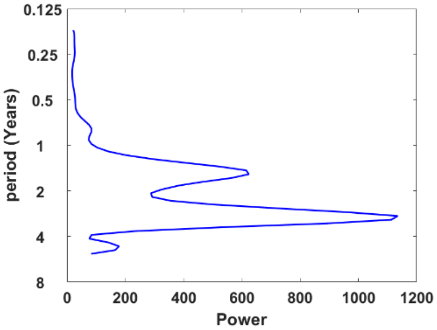

Cross Wavelet Transform (XWT) Left Panel (9c): XWT, Right Panel (9d): Wavelet power of XWT

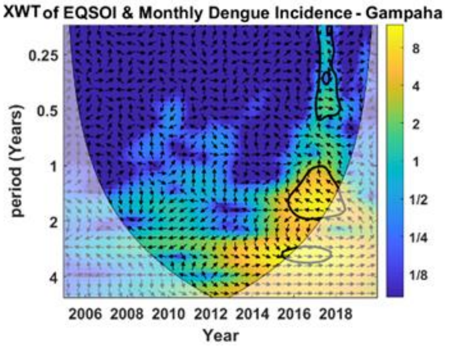

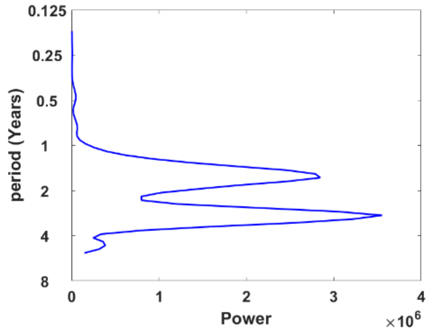

Cross Coherence (WTC) Left Panel (9e): WTC, Right Panel (9f): Wavelet power of WTC

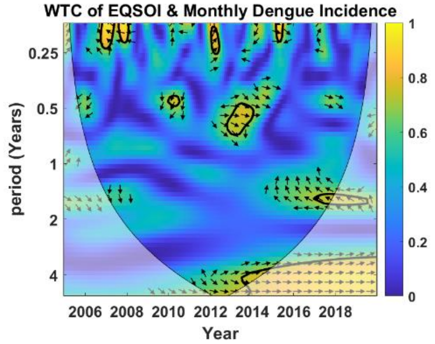

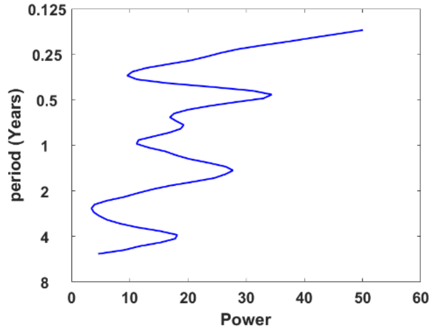

Reconstructed time series for selected period (9g)

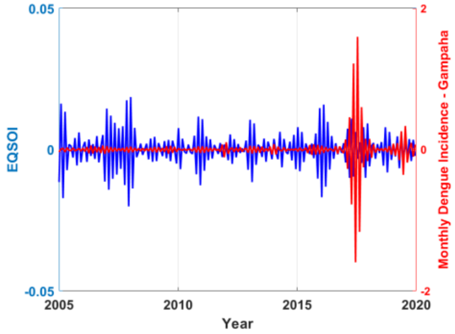

Figure 9: Wavelet analysis results of monthly EQSOI vs. dengue incidence of Gampaha District for 2004-2019: Panel 9a- continuous wavelet transform (CWT) variations; Panel 9b- wavelet power of CWT; Panel 9c- cross wavelet transform (XWT) variations; Panel 9d- wavelet power of XWT; Panel 9e- wavelet coherence (WTC); Panel 9f- wavelet power of WTC; Panel 9g- reconstructed time series for 2005-2019.

Figure 10: Wavelet analysis results of monthly MEI vs. dengue incidence of Gampaha District for 2004-2019.

Continuous Wavelet Transform (CWT) of monthly MEI Left Panel (10a): CWT, Right Panel (10b): Wavelet power

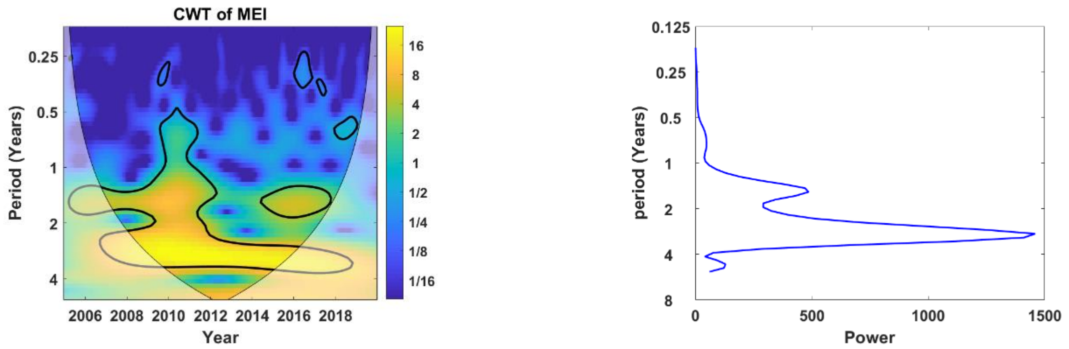

Cross Wavelet Transform (XWT) Left Panel (10c): XWT, Right Panel (10d): Wavelet power of XWT

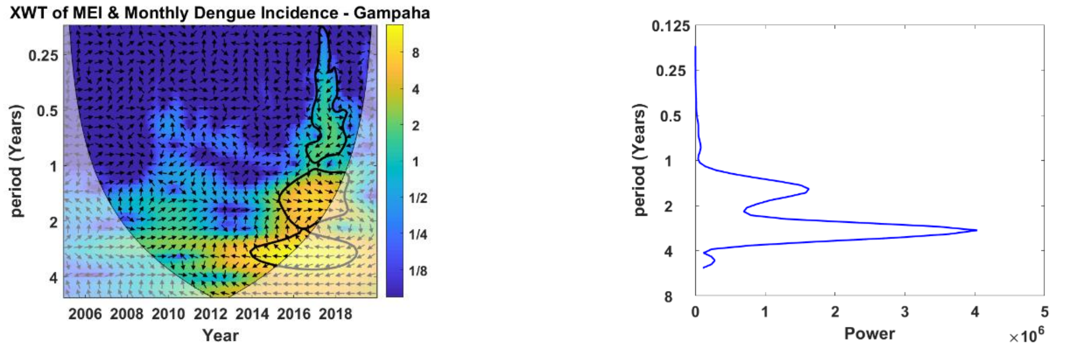

Cross Coherence (WTC) Left Panel (10e): WTC, Right Panel (10f): Wavelet power of WTC

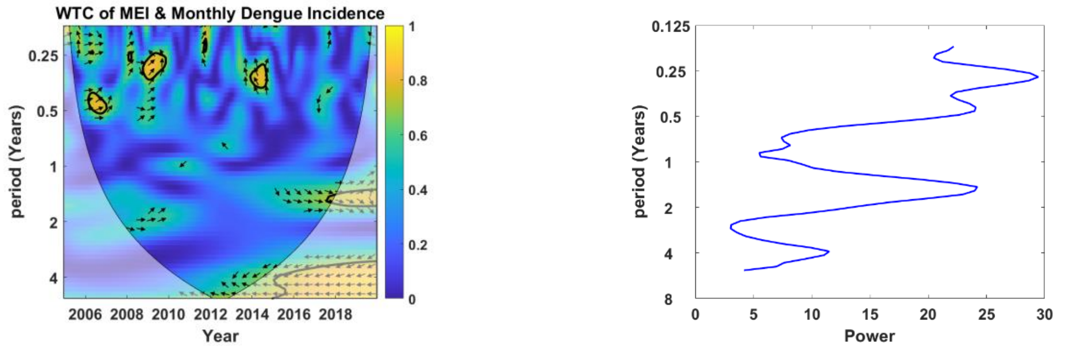

Reconstructed time series for selected period(10g)

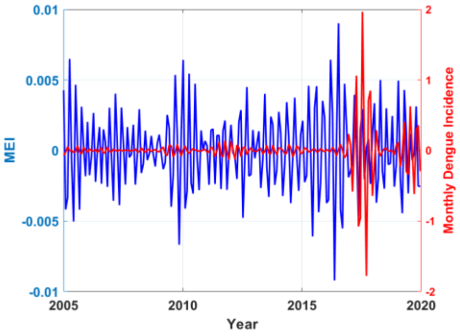

Figure 10: Wavelet analysis results of monthly MEI vs. dengue incidence of Gampaha District for 2004-2019: Panel 10a- continuous wavelet transform (CWT) variations; Panel 10b- wavelet

power of CWT; Panel 10c- cross wavelet transform (XWT) variations; Panel 10d- wavelet power of XWT; Panel 10e- wavelet coherence (WTC); Panel 10f- wavelet power of WTC; Panel 10g- reconstructed time series for 2005-2019.

Figure 11: Wavelet analysis results of monthly DMI vs. dengue incidence of Gampaha District for 2004-2019.

Continuous Wavelet Transform (CWT) of monthly DMI Left Panel (11a): CWT, Right Panel (11b): Wavelet power

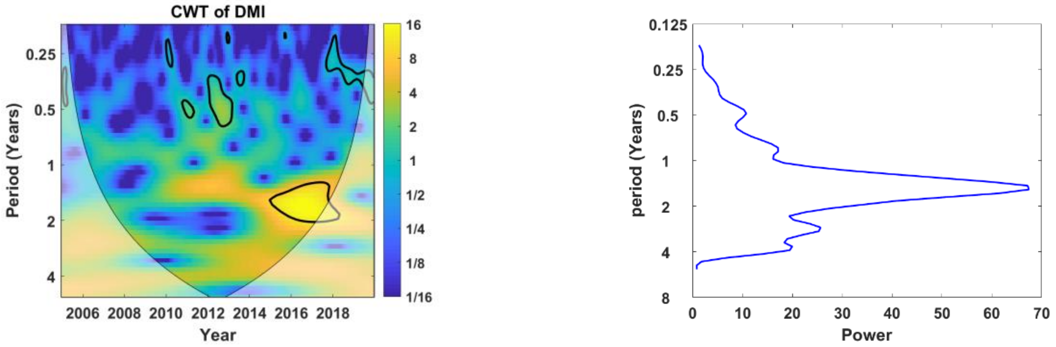

Cross Wavelet Transform (XWT) Left Panel (11c): XWT, Right Panel (11d): Wavelet power of XWT

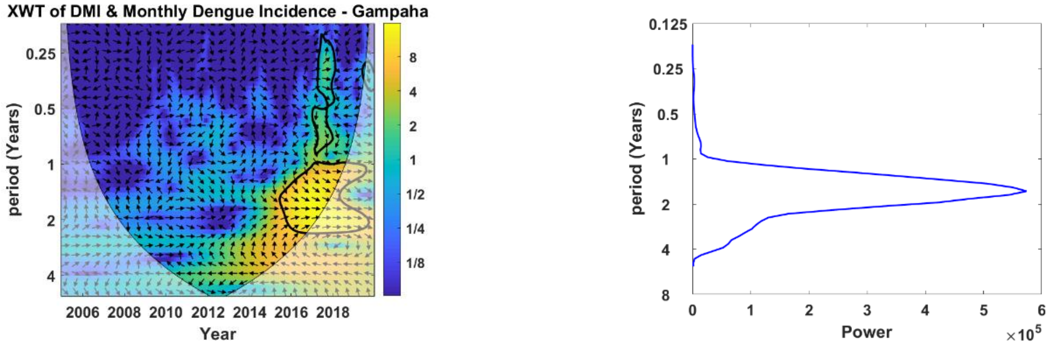

Cross Coherence (WTC) Left Panel (11e): WTC, Right Panel (11f): Wavelet power of WTC

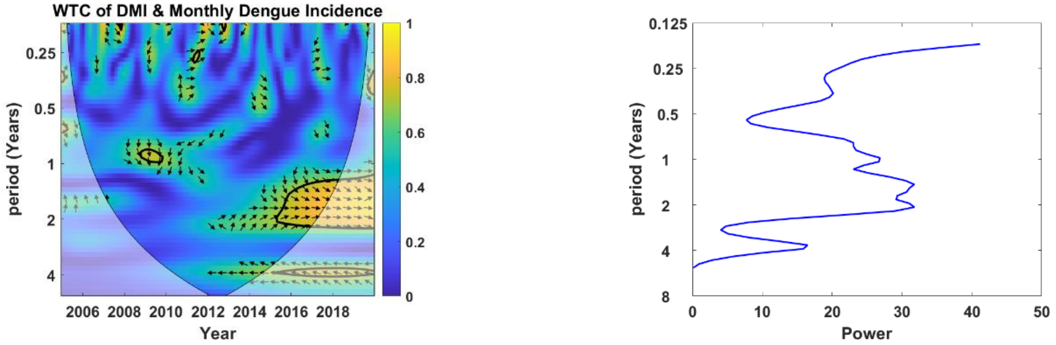

Reconstructed time series for selected period (11g)

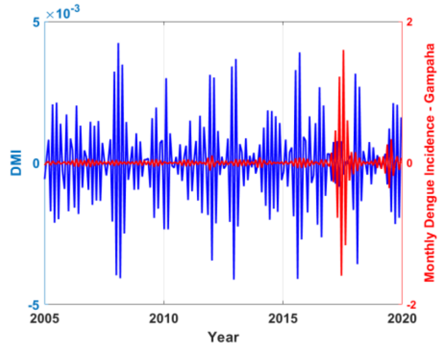

Figure 11: Wavelet analysis results of monthly EMI vs. dengue incidence of Gampaha District for 2004-2019: Panel 11a- continuous wavelet transform (CWT) variations; Panel 11b- wavelet power of CWT; Panel 11c- cross wavelet transform (XWT) variations; Panel 11d- wavelet power of XWT; Panel 11e- wavelet coherence (WTC); Panel 11f- wavelet power of WTC; Panel 11g- reconstructed time series for 2005-2019.

Figure 12: Wavelet analysis results of monthly EMI vs. dengue incidence of Gampaha District for 2004-2019.

Continuous Wavelet Transform (CWT) of monthly EMI Left Panel (12a): CWT, Right Panel (12b): Wavelet power

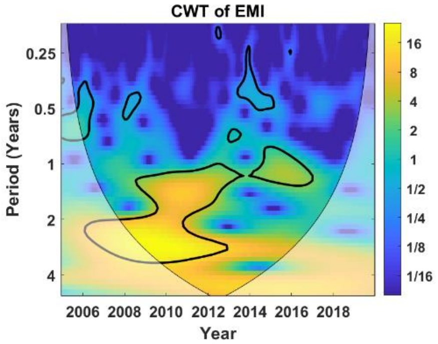

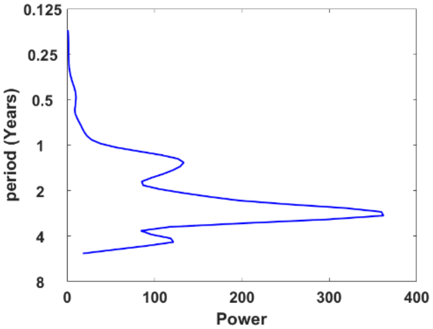

Cross Wavelet Transform (XWT) Left Panel (12c): XWT, Right Panel (12d): Wavelet power of XWT

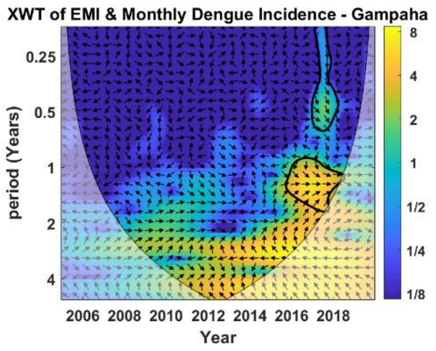

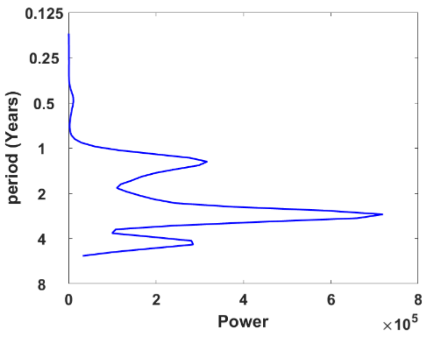

Cross Coherence (WTC) Left Panel (12e): WTC, Right Panel (12f): Wavelet power of WTC

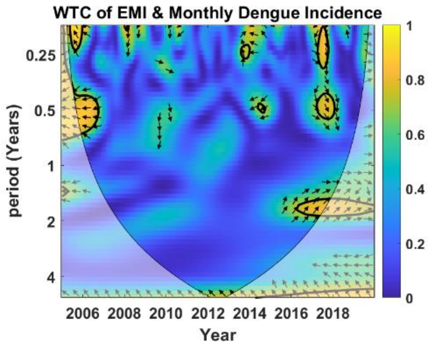

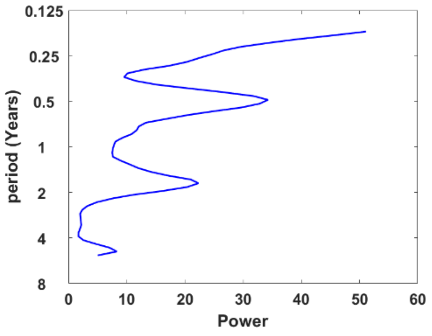

Reconstructed time series for selected period(12g)

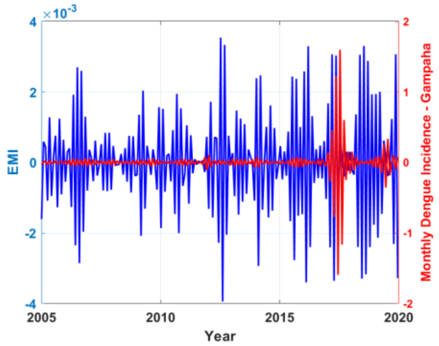

Figure 12: Wavelet analysis results of monthly EMI vs. dengue incidence of Gampaha District for 2004-2019: Panel 12a- continuous wavelet transform (CWT) variations; Panel 12b- wavelet power of CWT; Panel 12c- cross wavelet transform (XWT) variations; Panel 12d- wavelet power of XWT; Panel 12e- wavelet coherence (WTC); Panel 12f- wavelet power of WTC; Panel 12g- reconstructed time series for 2005-2019.

………………………………………………………………………………

Figure 13: Wavelet analysis results of monthly NINO 4 SSTA vs. dengue incidence of Kalutara District for 2004-2019.

Continuous Wavelet Transform (CWT) of monthly NINO 4 SSTA Left Panel (13a): CWT, Right Panel (13b): Wavelet power

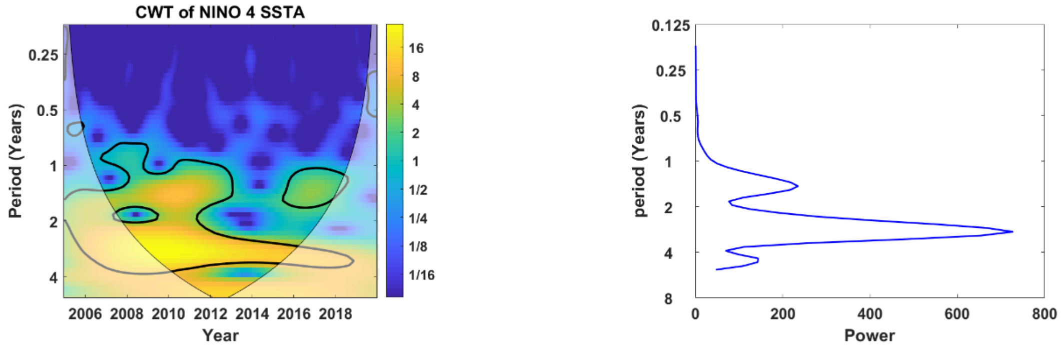

Cross Wavelet Transform (XWT) Left Panel (13c): XWT, Right Panel (13d): Wavelet power of XWT

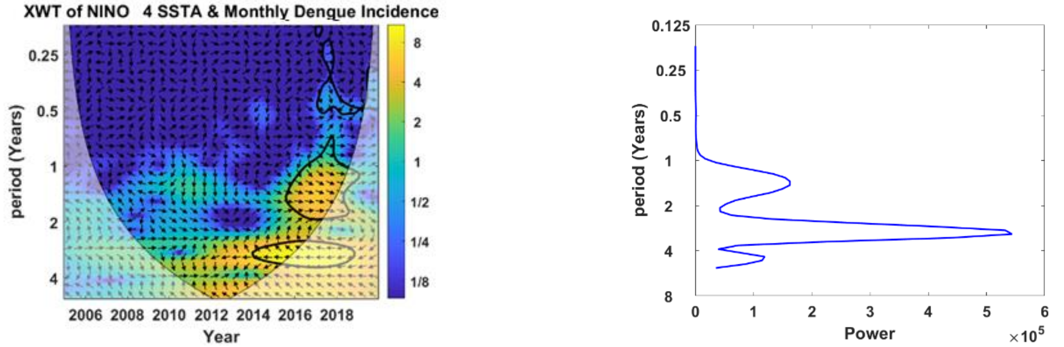

Cross Coherence (WTC) Left Panel (13e): WTC, Right Panel (13f): Wavelet power of WTC

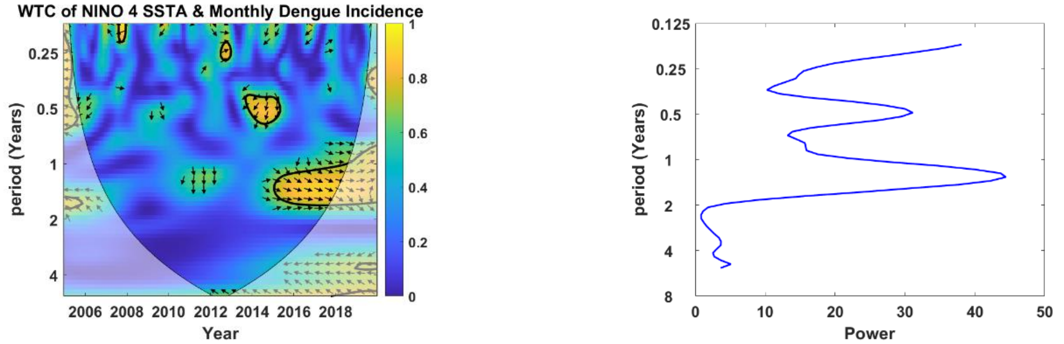

Reconstructed time series for selected period (13g)

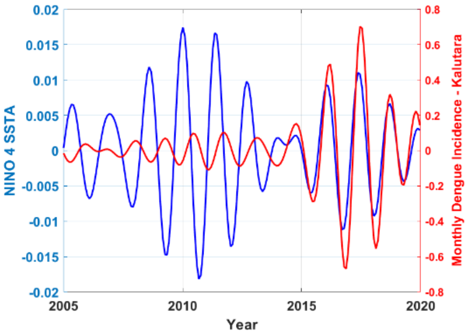

Figure 13: Wavelet analysis results of monthly NINO 4 SSTA vs. dengue incidence of Kalutara District for 2004-2019: Panel 13a- continuous wavelet transform (CWT) variations; Panel 13b-

wavelet power of CWT; Panel 13c- cross wavelet transform (XWT) variations; Panel 13d- wavelet power of XWT; Panel 13e- wavelet coherence (WTC); Panel 13f- wavelet power of WTC; Panel 13g- reconstructed time series for 2005-2019.

Figure 14: Wavelet analysis results of monthly NINO 3.4 SSTA vs. dengue incidence of Kalutara District for 2004-2019.

Continuous Wavelet Transform (CWT) of monthly NINO3.4 SSTA Left Panel (14a): CWT, Right Panel (14b): Wavelet power

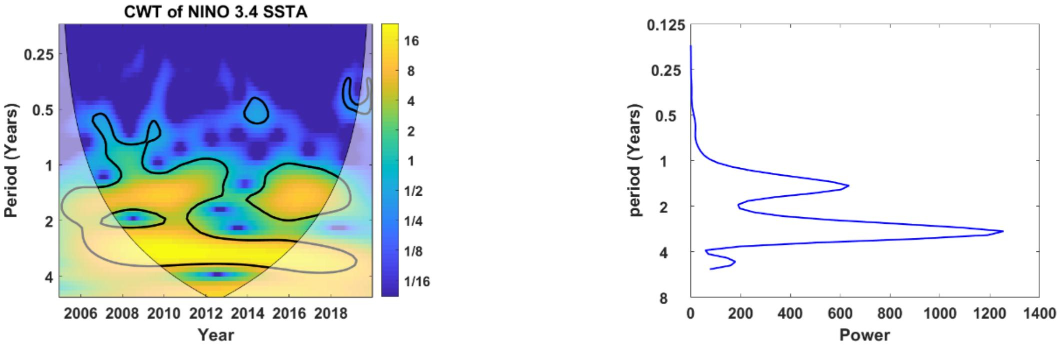

Cross Wavelet Transform (XWT) Left Panel (14c): XWT, Right Panel (14d): Wavelet power of XWT

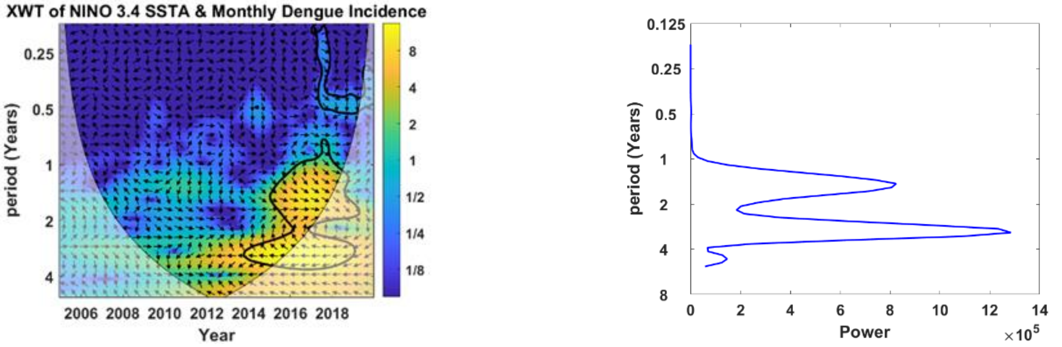

Cross Coherence (WTC) Left Panel (14e): WTC, Right Panel (14f): Wavelet power of WTC

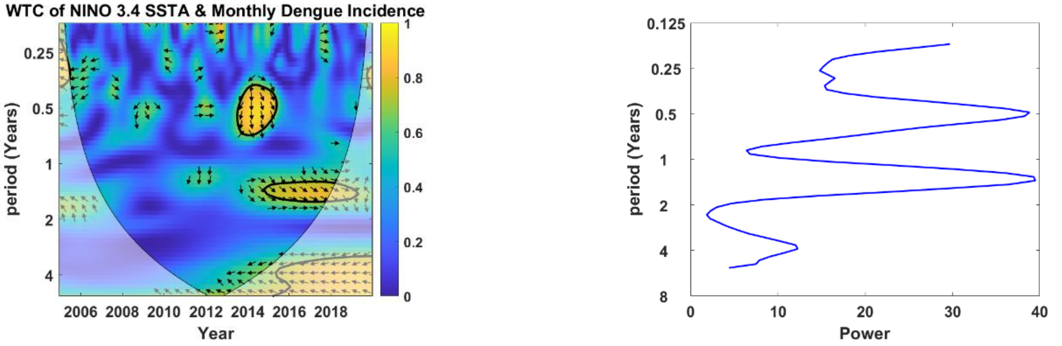

Reconstructed time series for selected period(14g)

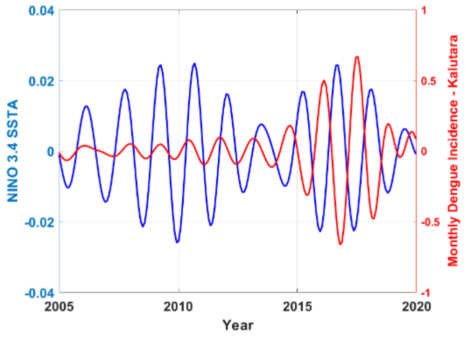

Figure 14: Wavelet analysis results of monthly NINO 3.4 SSTA vs. dengue incidence of Kalutara District for 2004-2019: Panel 14a- continuous wavelet transform (CWT) variations; Panel 14b- wavelet power of CWT; Panel 3c- cross wavelet transform (XWT) variations; Panel 14d- wavelet power of XWT; Panel 14e- wavelet coherence (WTC); Panel 14f- wavelet power of WTC; Panel 14g- reconstructed time series for 2005-2019.

Figure 15: Wavelet analysis results of monthly EQSOI vs. dengue incidence of Kalutara District for 2004-2019.

Continuous Wavelet Transform (CWT) of monthly EQSOI Left Panel (15a): CWT, Right Panel (15b): Wavelet power

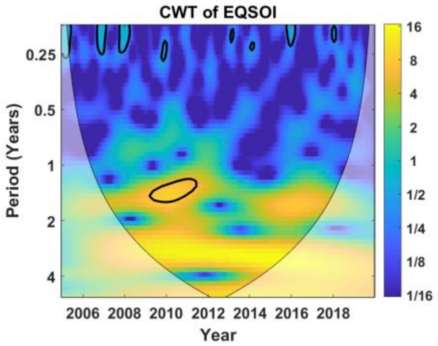

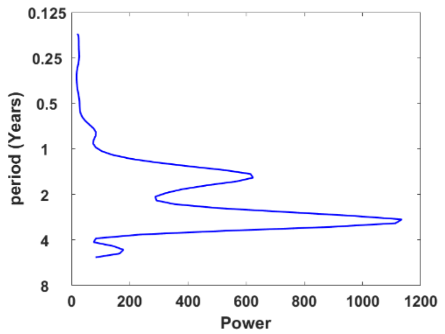

Cross Wavelet Transform (XWT) Left Panel (15c): XWT, Right Panel (15d): Wavelet power of XWT

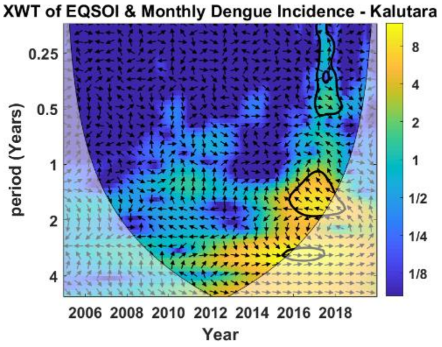

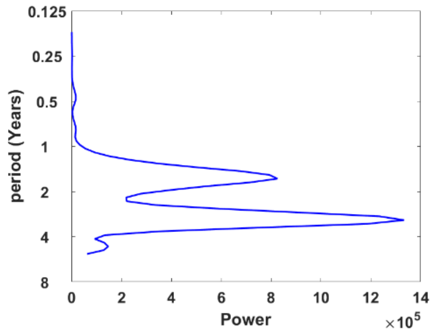

Cross Coherence (WTC) Left Panel (15e): WTC, Right Panel (15f): Wavelet power of

WTC

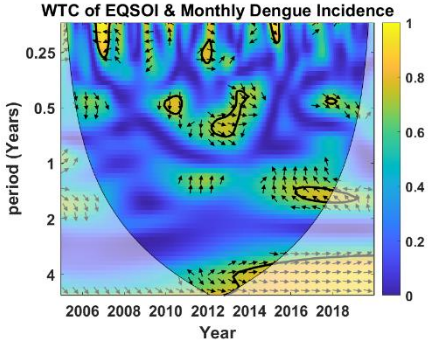

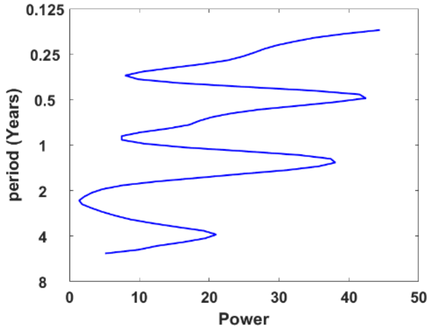

Reconstructed time series for selected period(15g)

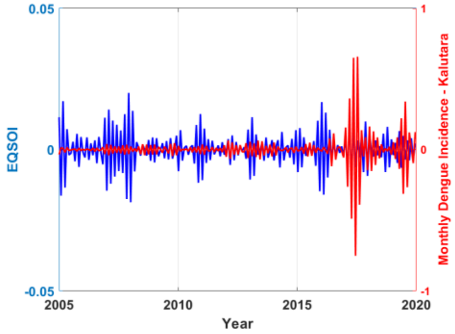

Figure 15: Wavelet analysis results of monthly EQSOI vs. dengue incidence of Kalutara District for 2004-2019: Panel 15a- continuous wavelet transform (CWT) variations; Panel 15b- wavelet power of CWT; Panel 15c- cross wavelet transform (XWT) variations; Panel 15d- wavelet power of XWT; Panel 15e- wavelet coherence (WTC); Panel 15f- wavelet power of WTC; Panel 15g- reconstructed time series for 2005-2019.

Figure 16: Wavelet analysis results of monthly MEI vs. dengue incidence of Kalutara District for 2004-2019.

Continuous Wavelet Transform (CWT) of monthly MEI Left Panel (16a): CWT, Right Panel (16b): Wavelet power

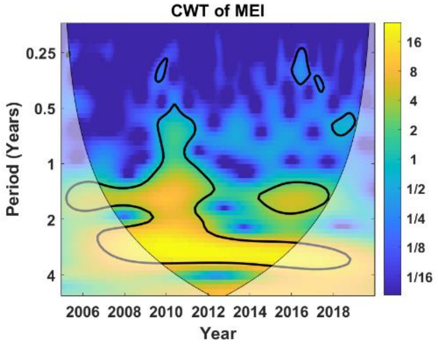

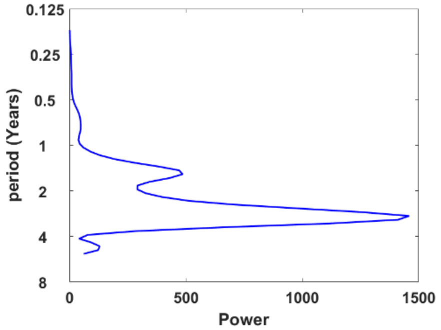

Cross Wavelet Transform (XWT) Left Panel (16c): XWT, Right Panel (16d): Wavelet

power of XWT

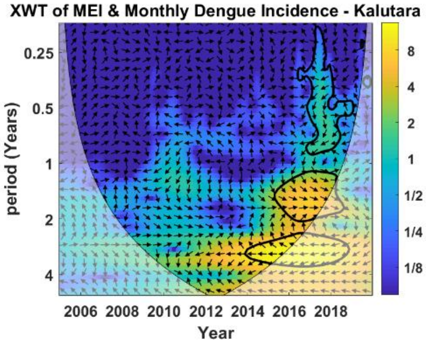

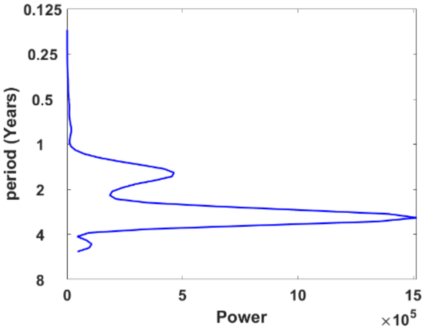

Cross Coherence (WTC) Left Panel (16e): WTC, Right Panel (16f): Wavelet power of WTC

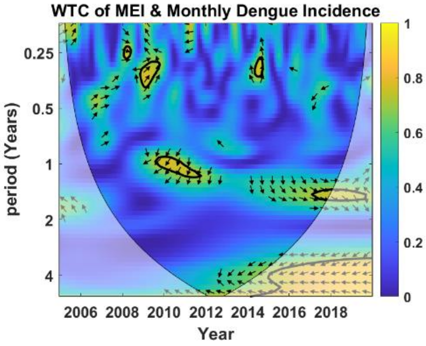

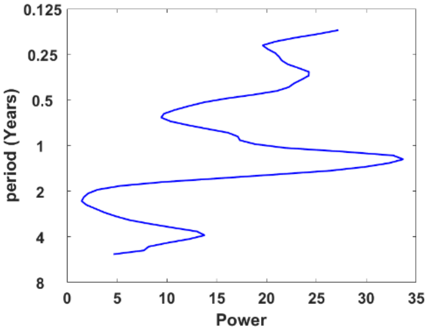

Reconstructed time series for selected period (16g)

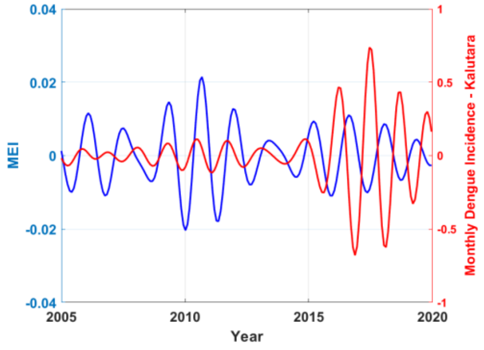

Figure 16: Wavelet analysis results of monthly MEI vs. dengue incidence of Kalutara District for 2004-2019: Panel 16a- continuous wavelet transform (CWT) variations; Panel 16b- wavelet power of CWT; Panel 16c- cross wavelet transform (XWT) variations; Panel 16d- wavelet power of XWT; Panel 16e- wavelet coherence (WTC); Panel 16f- wavelet power of WTC; Panel 16g- reconstructed time series for 2005-2019.

Figure 17: Wavelet analysis results of monthly DMI vs. dengue incidence of Kalutara District for 2004-2019.

Continuous Wavelet Transform (CWT) of monthly DMI Left Panel (17a): CWT, Right

Panel (17b): Wavelet power

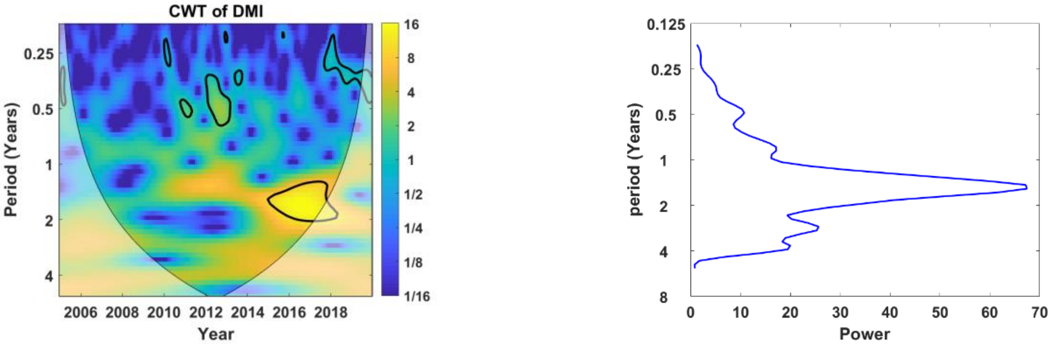

Cross Wavelet Transform (XWT) Left Panel (17c): XWT, Right Panel (17d): Wavelet power of XWT

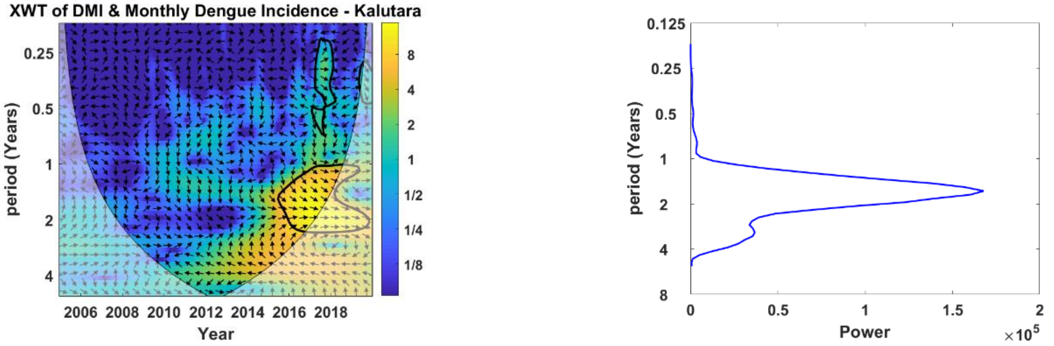

Cross Coherence (WTC) Left Panel (17e): WTC, Right Panel (17f): Wavelet power of WTC

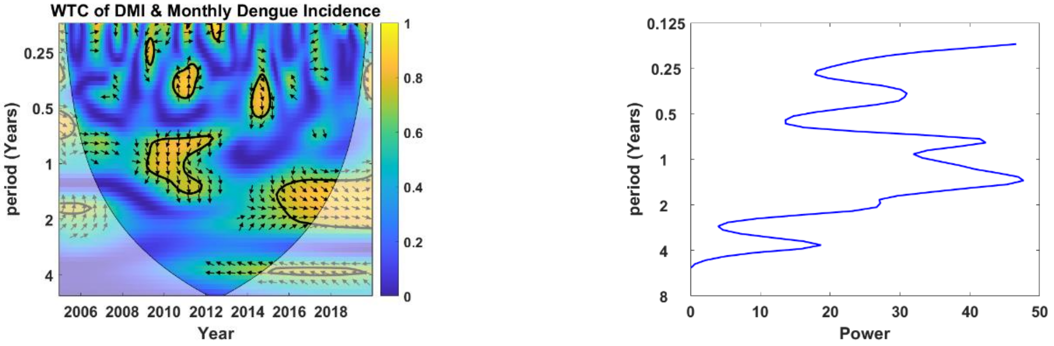

Reconstructed time series for selected period(17g)

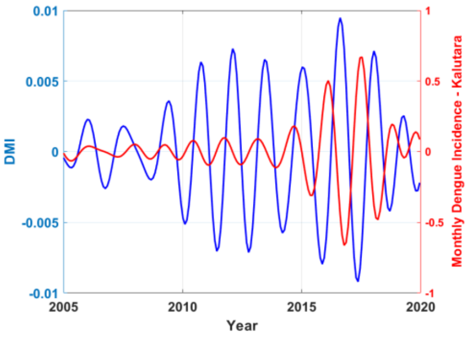

Figure 17: Wavelet analysis results of monthly DMI vs. dengue incidence of Kalutara District for 2004-2019: Panel 17a- continuous wavelet transform (CWT) variations; Panel 17b- wavelet power of CWT; Panel 17c- cross wavelet transform (XWT) variations; Panel 17d- wavelet power of XWT; Panel 17e- wavelet coherence (WTC); Panel 17f- wavelet power of WTC; Panel 17g- reconstructed time series for 2005-2019.

Figure 18: Wavelet analysis results of monthly EMI vs. dengue incidence of Kalutara District for 2004-2019.

Continuous Wavelet Transform (CWT) of monthly EMI Left Panel (18a): CWT, Right Panel (18b): Wavelet power

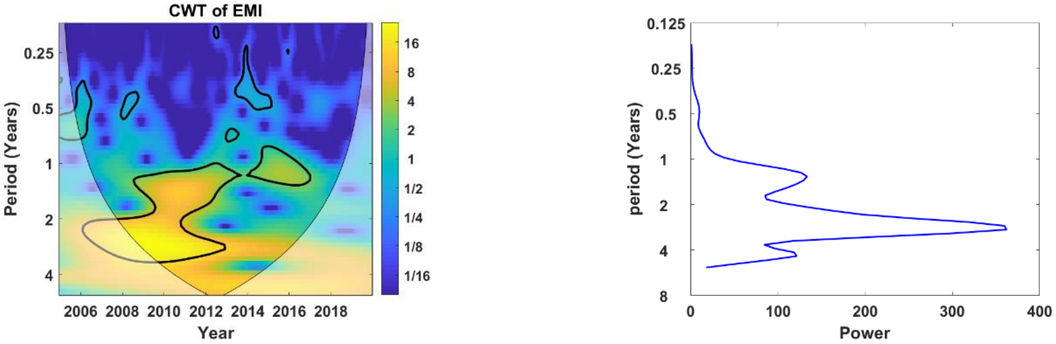

Cross Wavelet Transform (XWT) Left Panel (3c): XWT, Right Panel (18d): Wavelet power of XWT

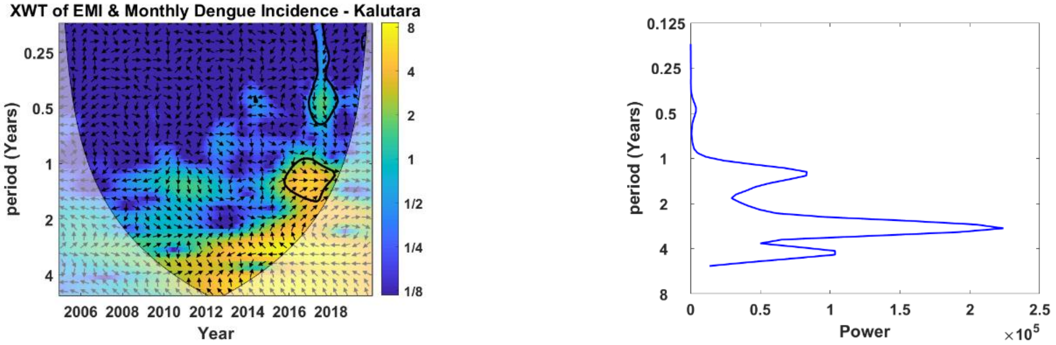

Cross Coherence (WTC) Left Panel (18e): WTC, Right Panel (18f): Wavelet power of WTC

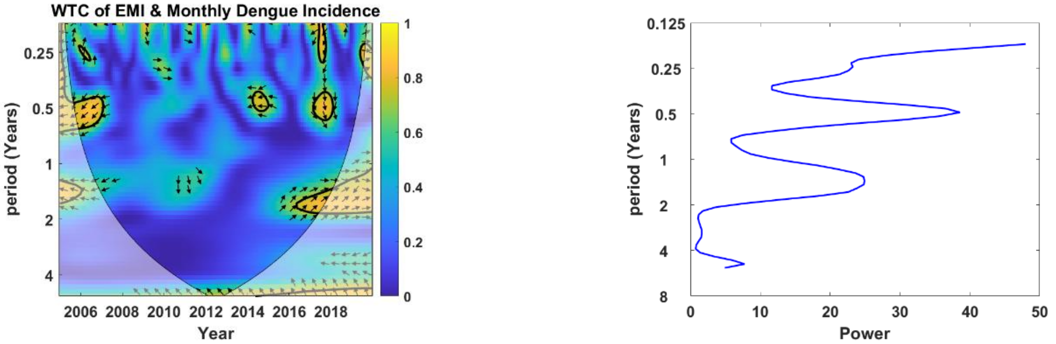

Reconstructed time series for selected period (18g)

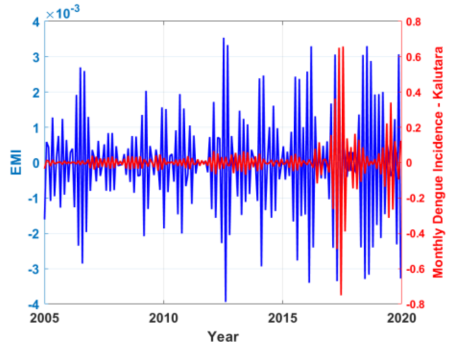

Figure 18: Wavelet analysis results of monthly EMI vs. dengue incidence of Kalutara District for 2004-2019: Panel 18a- continuous wavelet transform (CWT) variations; Panel 18b- wavelet power of CWT; Panel 18c- cross wavelet transform (XWT) variations; Panel 18d- wavelet power of XWT; Panel 18e- wavelet coherence (WTC); Panel 18f- wavelet power of WTC; Panel 18g- reconstructed time series for 2005-2019.

……………………………………………………………………………………………..

Figure 19: Wavelet analysis results of monthly NINO4 vs. dengue incidence of Western Province for 2004-2019.

Continuous Wavelet Transform (CWT) of monthly NINO4 SSTA Left Panel (19a): CWT, Right Panel (19b): Wavelet power

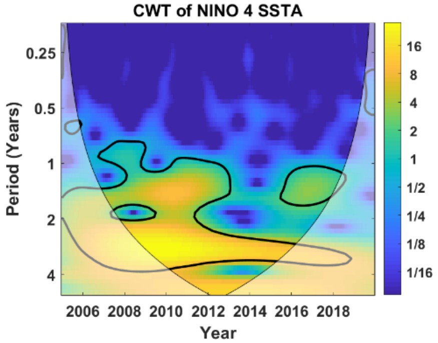

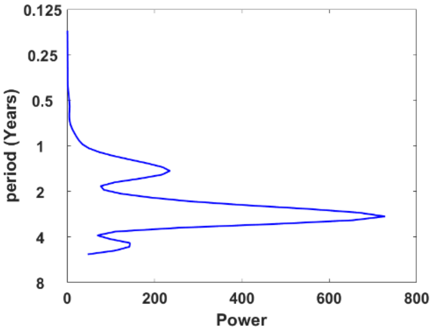

Cross Wavelet Transform (XWT) Left Panel (19c): XWT, Right Panel (19d): Wavelet power of XWT

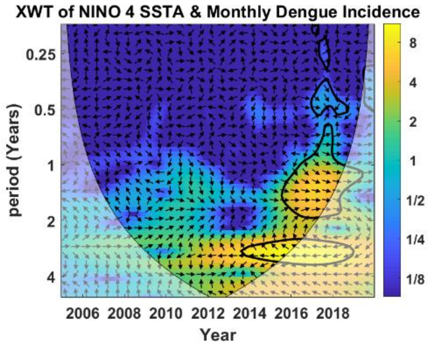

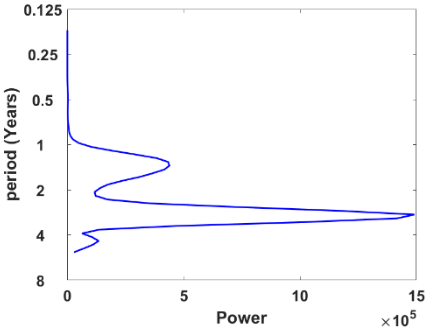

Cross Coherence (WTC) Left Panel (19e): WTC, Right Panel (19f): Wavelet power of WTC

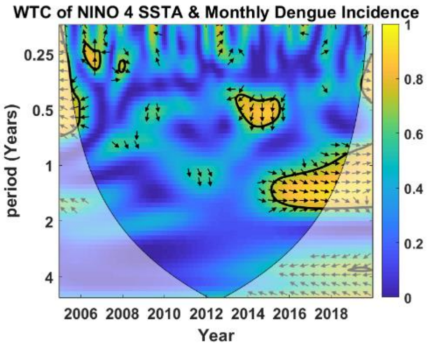

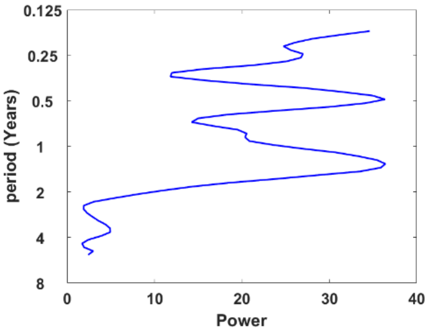

Reconstructed time series for selected period(19g)

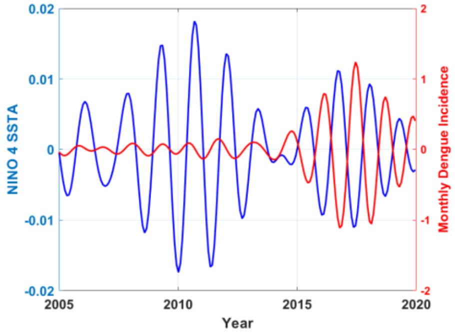

Figure 19: Wavelet analysis results of monthly NINO4 vs. dengue incidence of Western Province for 2004-2019: Panel 19a- continuous wavelet transform (CWT) variations; Panel 19b- wavelet power of CWT; Panel 19c- cross wavelet transform (XWT) variations; Panel 19d- wavelet power of XWT; Panel 19e- wavelet coherence (WTC); Panel 19f- wavelet power of WTC; Panel 19g- reconstructed time series for 2005-2019.

Figure 20: Wavelet analysis results of monthly NINO 3.4 vs. dengue incidence of Western Province for 2004-2019.

Continuous Wavelet Transform (CWT) of monthly NINO3.4 SSTA Left Panel (20a): CWT, Right Panel (20b): Wavelet power

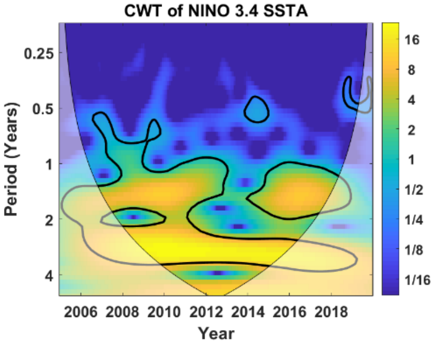

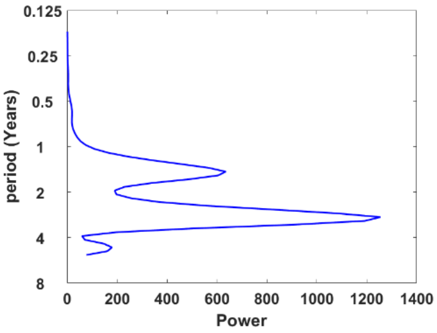

Cross Wavelet Transform (XWT) Left Panel (20c): XWT, Right Panel (20d): Wavelet power of XWT

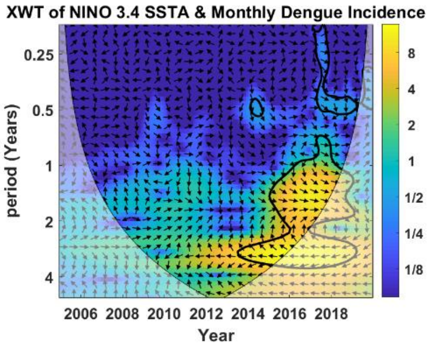

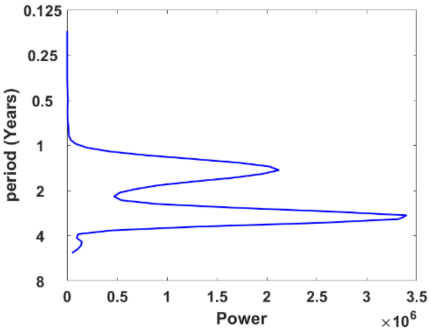

Cross Coherence (WTC) Left Panel (20e): WTC, Right Panel (20f): Wavelet power of WTC

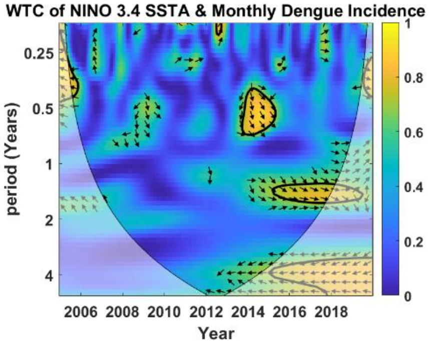

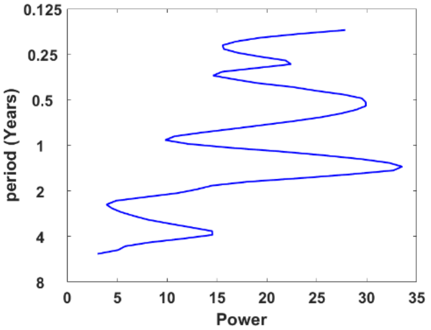

Reconstructed time series for selected period(20g)

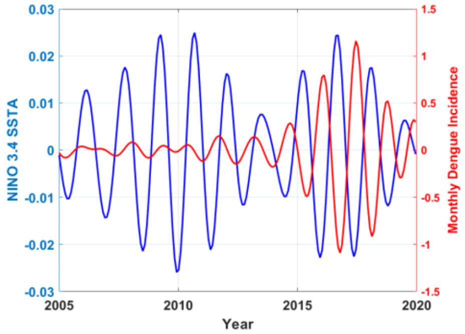

Figure 20: Wavelet analysis results of monthly NINO 3.4 vs. dengue incidence of Western Province for 2004-2019: Panel 20a- continuous wavelet transform (CWT) variations; Panel 20b- wavelet power of CWT; Panel 20c- cross wavelet transform (XWT) variations; Panel 20d- wavelet power of XWT; Panel 20e- wavelet coherence (WTC); Panel 20f- wavelet power of WTC; Panel 20g- reconstructed time series for 2005-2019.

Figure 21: Wavelet analysis results of monthly EQSOI vs. dengue incidence of Western Province for 2004-2019.

Continuous Wavelet Transform (CWT) of monthly EQSOI Left Panel (21a): CWT, Right Panel (21b): Wavelet power

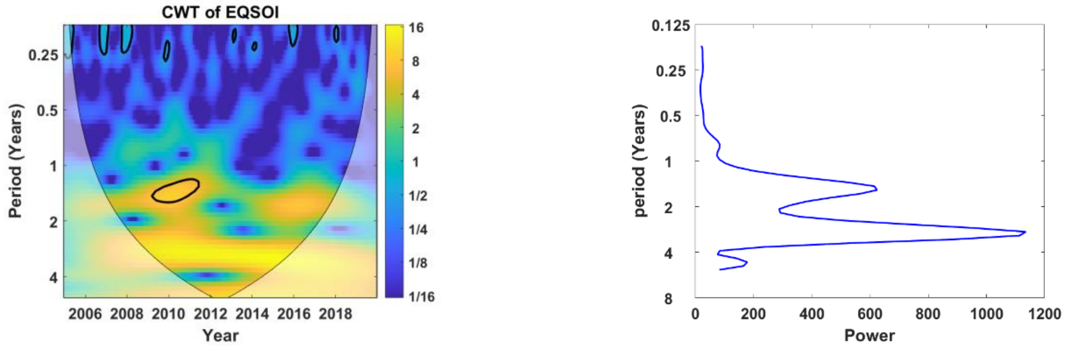

Cross Wavelet Transform (XWT) Left Panel (21c): XWT, Right Panel (21d): Wavelet power of XWT

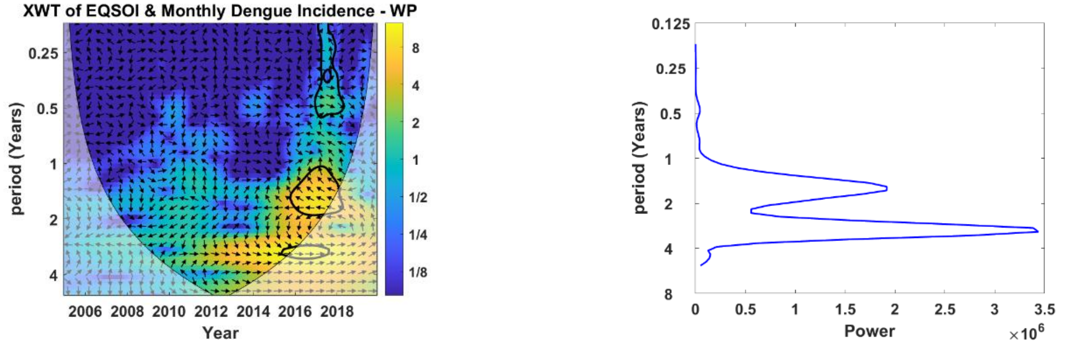

Cross Coherence (WTC) Left Panel (21e): WTC, Right Panel (21f): Wavelet power of WTC

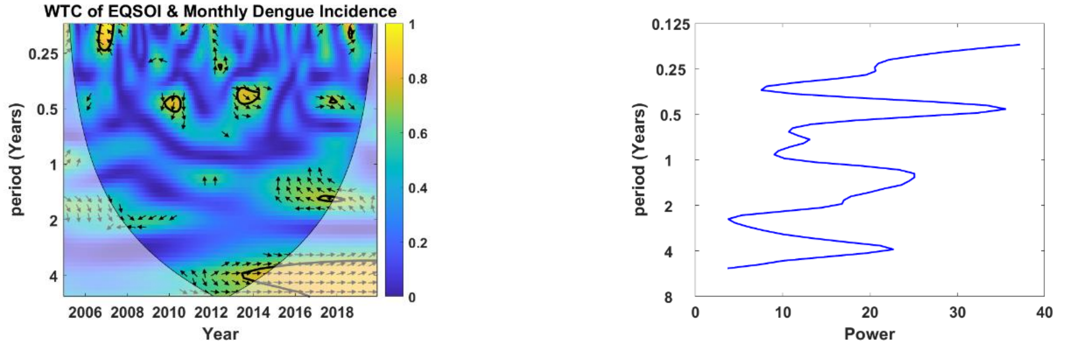

Reconstructed time series for selected period (21g)

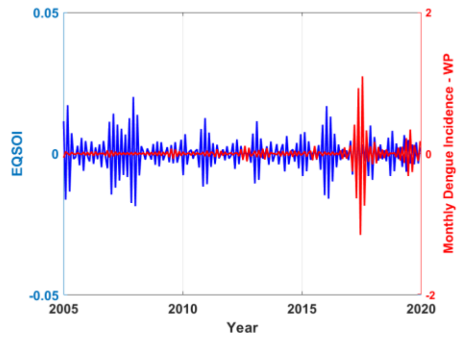

Figure 21: Wavelet analysis results of monthly EQSOI vs. dengue incidence of Western Province for 2004-2019: Panel 21a- continuous wavelet transform (CWT) variations; Panel 21b- wavelet

power of CWT; Panel 21c- cross wavelet transform (XWT) variations; Panel 21d- wavelet power of XWT; Panel 21e- wavelet coherence (WTC); Panel 21f- wavelet power of WTC; Panel 21g- reconstructed time series for 2005-2019.

Figure 22: Wavelet analysis results of monthly MEI vs. dengue incidence of Western Province for 2004-2019.

Continuous Wavelet Transform (CWT) of monthly MEI Left Panel (22a): CWT, Right Panel (22b): Wavelet power

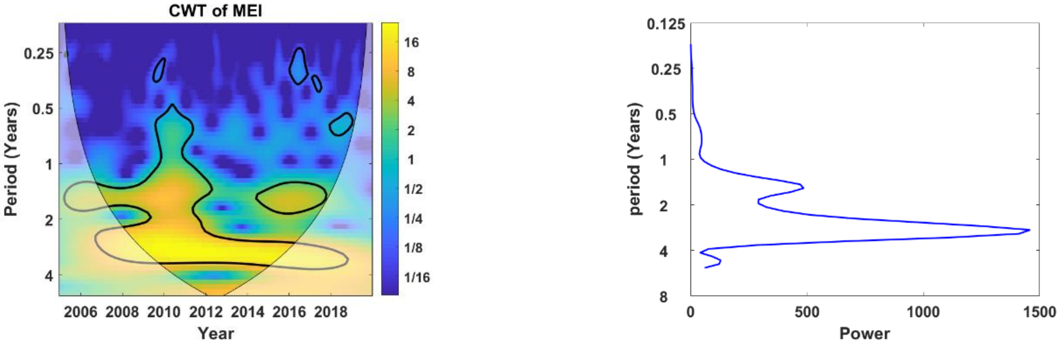

Cross Wavelet Transform (XWT) Left Panel (22c): XWT, Right Panel (22d): Wavelet power of XWT

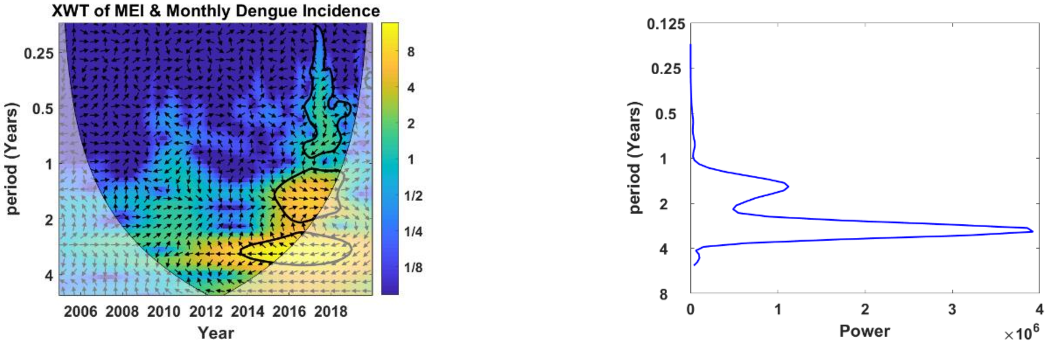

Cross Coherence (WTC) Left Panel (22e): WTC, Right Panel (22f): Wavelet power of WTC

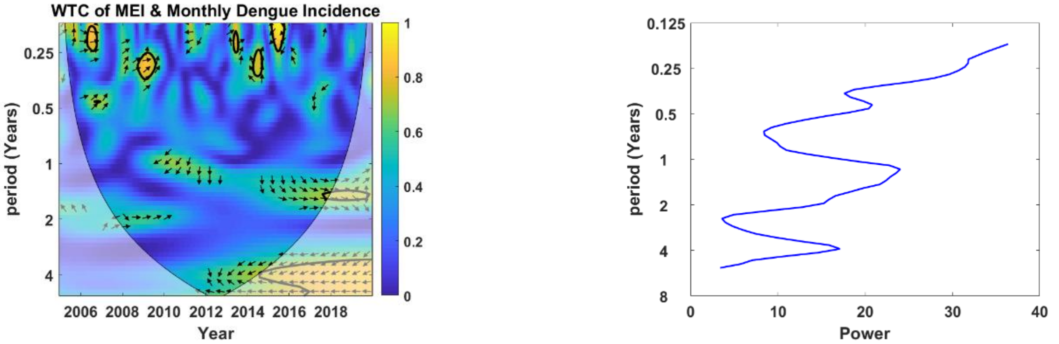

Reconstructed time series for selected period (22g)

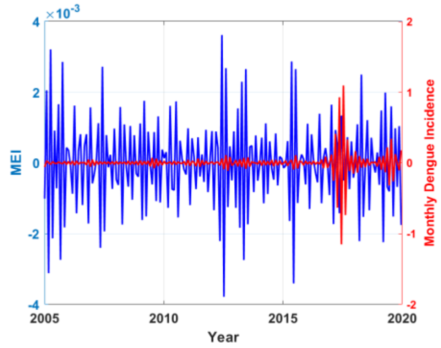

Figure 22: Wavelet analysis results of monthly MEI vs. dengue incidence of Western Province for 2004-2019: Panel 22a- continuous wavelet transform (CWT) variations; Panel 22b- wavelet power of CWT; Panel 22c- cross wavelet transform (XWT) variations; Panel 22d- wavelet power of XWT; Panel 22e- wavelet coherence (WTC); Panel 22f- wavelet power of WTC; Panel 22g- reconstructed time series for 2005-2019.

Figure 23: Wavelet analysis results of monthly DMI vs. dengue incidence of Western Province for 2004-2019.

Continuous Wavelet Transform (CWT) of monthly DMI Left Panel (23a): CWT, Right Panel (23b): Wavelet power

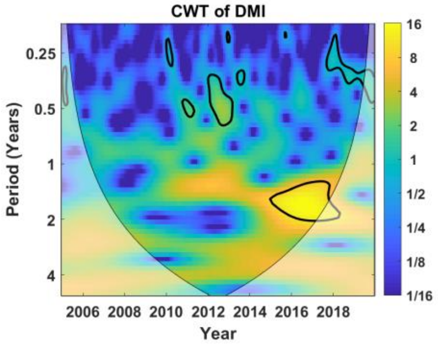

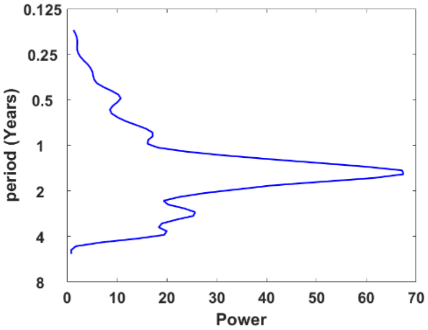

Cross Wavelet Transform (XWT) Left Panel (23c): XWT, Right Panel (23d): Wavelet power of XWT

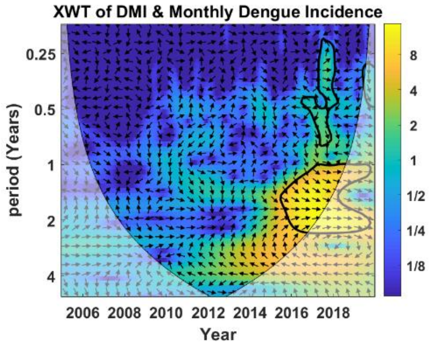

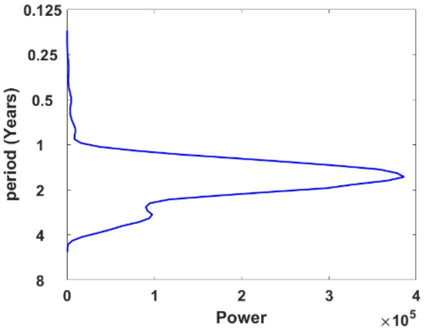

Cross Coherence (WTC) Left Panel (23e): WTC, Right Panel (23f): Wavelet power of WTC

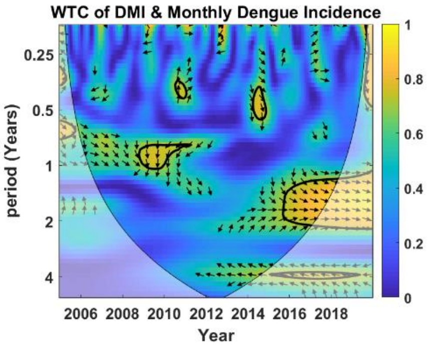

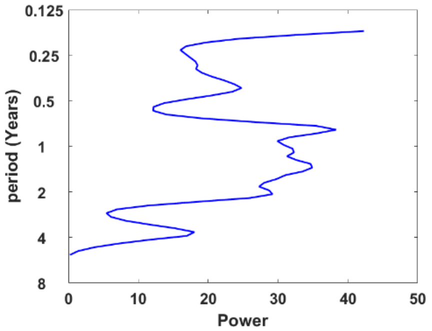

Reconstructed time series for selected period(23g)

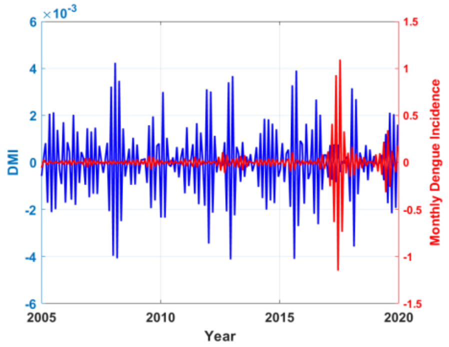

Figure 23: Wavelet analysis results of monthly MEI vs. dengue incidence of Western Province for 2004-2019: Panel 23a- continuous wavelet transform (CWT) variations; Panel 23b- wavelet power of CWT; Panel 23c- cross wavelet transform (XWT) variations; Panel 23d- wavelet power of XWT; Panel 23e- wavelet coherence (WTC); Panel 23f- wavelet power of WTC; Panel 23g- reconstructed time series for 2005-2019.

Figure 24: Wavelet analysis results of monthly EMI vs. dengue incidence of Western Province for 2004-2019.

Continuous Wavelet Transform (CWT) of monthly EMI Left Panel (24a): CWT, Right Panel (24b): Wavelet power

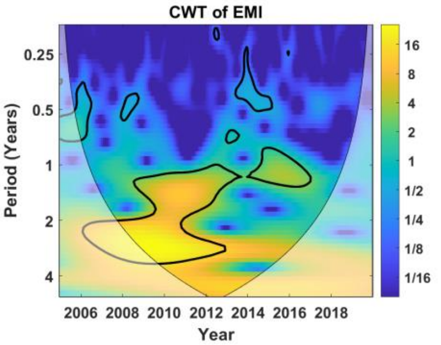

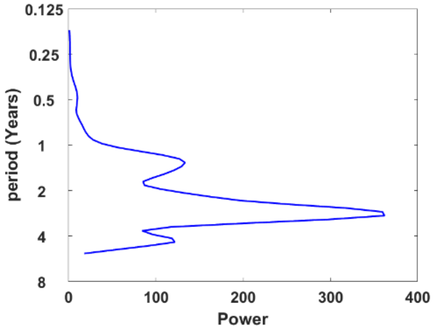

Cross Wavelet Transform (XWT) Left Panel (24c): XWT, Right Panel (24d): Wavelet power of XWT

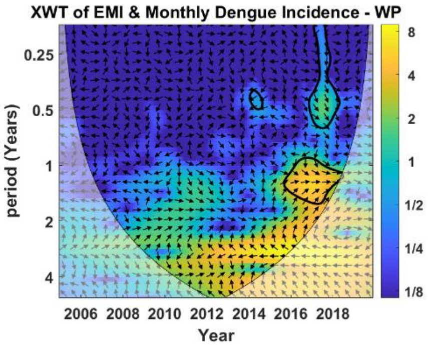

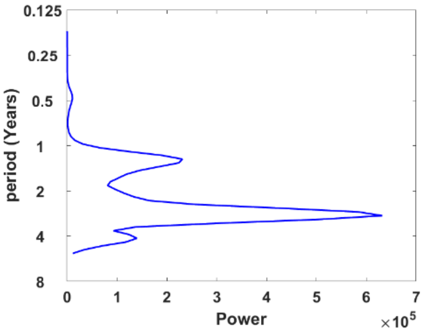

Cross Coherence (WTC) Left Panel (24e): WTC, Right Panel (24f): Wavelet power of WTC

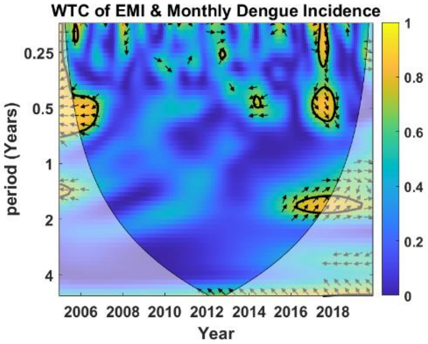

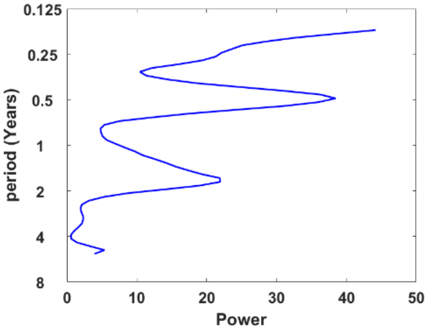

Reconstructed time series for selected period(24g)

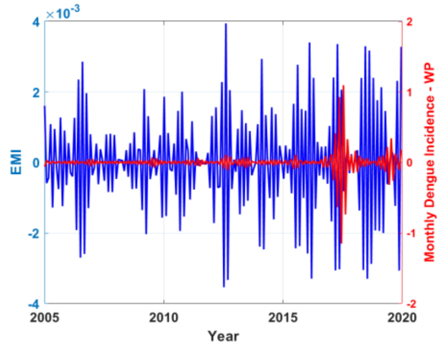

Figure 24: Wavelet analysis results of monthly EMI vs. dengue incidence of Western Province for 2004-2019: Panel 24a- continuous wavelet transform (CWT) variations; Panel 24b- wavelet power of CWT; Panel 24c- cross wavelet transform (XWT) variations; Panel 24d- wavelet power of XWT; Panel 24e- wavelet coherence (WTC); Panel 24f- wavelet power of WTC; Panel 24g- reconstructed time series for 2005-2019.

Table1: The summary of wavelet analysis results- Teleconnections vs WP Dengue

District	Nino 4	Nino3.4	EQSOI	MEI	DMI	EMI (Modoki)

**Colombo District**	DI peaks after 6.1 (0 to 9) months of Nino4 troughs	1.DI peaks after 3.4 (0 to 7)months of Nino3.4 peaks , 2.DI peaks after 6.2 (0 to 10) months of Nino3.4 troughs	1. DI peaks after 3.2 months (0-5 months) of EQSOI troughs. 2. DI peaks after 1.3 months (0-3 months) of EQSOI peaks.	No correlation	DI peaks after 1.2 (0 to 3) months of DMI troughs	DI peaks after 2.4 months (1-4 months) of EMI peaks
**Gampaha District**	1.DI peaks after 1 month of Nino4 peaks 2.DI peaks after 1.9 (1 to 2) months of Nino4 troughs	1.DI peaks after 2 (1 to 4)months of Nino3.4 peaks , 2.DI peaks after 8.2 (1 to 13) months of Nino3.4 troughs	No correlation.	DI peaks after 6.1 (1 to 9)months of MEI peaks , DI peak after 4.4 (1 to 7) months of MEI troughs	No correlation	No correlation.
**Kalutara District**	DI peaks after 2.6 (0 to 5)months of Nino4 peaks	DI peaks after 4.7 (0 to 9)months of Nino3.4 peaks	DI peaks after 1 month (0-3 months) of EQSOI troughs.	DI peaks after 4 (1 to 7)months of MEI peaks	1.DI peaks after 4 (1 to 7)months of DMI peaks , 2. DI peaks after 9.8 (1 to 15) months of DMI troughs	No correlation
**Western Province (As a whole)**	1. DI peaks after about 1.6 months (0-2 months) of Nino 4 peaks 2. DI peaks after about 4.6 months (varies 3-5 months) of Nino 4 troughs	1. DI peaks after about 1.6 months (0-2 months) of Nino3.4 peaks 2. DI peaks after 4.7 months (3-6 months) of Nino 3.4 troughs	No correlation.	1. DI peaks after 4.9 months (0.3-9.6 months) of EMI peaks 2. DI peaks after 4.9 months (0. 3-9.8 months) of EMI troughs	No correlation	1. DI peaks after 4.9 months (0.3-9.6 months) of EMI peaks 2. DI peak after 4.9 months (0. 3-9.8 months) of EMI troughs
